# Translational Research on Bee Pollen as a Source of Nutrients: A Scoping Review from Bench to Real World

**DOI:** 10.3390/nu15102413

**Published:** 2023-05-22

**Authors:** Rachid Kacemi, Maria G. Campos

**Affiliations:** 1 Observatory of Drug-Herb Interactions, Laboratory of Pharmacognosy, Faculty of Pharmacy, University of Coimbra, Heath Sciences Campus, Azinhaga de Santa Comba, 3000-548 Coimbra, Portugal; kacemi@gmail.com; 2 Coimbra Chemistry Centre (CQC, FCT Unit 313), Faculty of Science and Technology, University of Coimbra, Rua Larga, 3004-516 Coimbra, Portugal

**Keywords:** antioxidant, dietary, disease recoveries, elderly, metabolic

## Abstract

The emphasis on healthy nutrition is gaining a forefront place in current biomedical sciences. Nutritional deficiencies and imbalances have been widely demonstrated to be involved in the genesis and development of many world-scale public health burdens, such as metabolic and cardiovascular diseases. In recent years, bee pollen is emerging as a scientifically validated candidate, which can help diminish conditions through nutritional interventions. This matrix is being extensively studied, and has proven to be a very rich and well-balanced nutrient pool. In this work, we reviewed the available evidence on the interest in bee pollen as a nutrient source. We mainly focused on bee pollen richness in nutrients and its possible roles in the main pathophysiological processes that are directly linked to nutritional imbalances. This scoping review analyzed scientific works published in the last four years, focusing on the clearest inferences and perspectives to translate cumulated experimental and preclinical evidence into clinically relevant insights. The promising uses of bee pollen for malnutrition, digestive health, metabolic disorders, and other bioactivities which could be helpful to readjust homeostasis (as it is also true in the case of anti-inflammatory or anti-oxidant needs), as well as the benefits on cardiovascular diseases, were identified. The current knowledge gaps were identified, along with the practical challenges that hinder the establishment and fructification of these uses. A complete data collection made with a major range of botanical species allows more robust clinical information.

## 1. Introduction

Substantial research has been conducted on the composition and pharmacological bioactivities of bee pollen (BP), indicating both its usefulness and safety. Numerous studies have shown that BP has a rich and well-balanced composition to serve as a human food and supplement, while its richness of bioactive compounds, especially polyphenols, confers to it a large array of biological and pharmacological activities [1]. BP-related risks may mainly emanate from external factors [2], or from improper storage and processing conditions [1]. Allergic reactions are rare and BP is perceived as a safe product in most physiological situations including in childhood, in older age, and in disease recoveries [3,4]. BP from different floral sources has been reported to possess anesthetic, anti-allergic, anti-androgen, anti-atherosclerotic, anti-cancer (anticarcinogenic and anti-mutagenic), anti-inflammatory, antimicrobial (antibacterial, antifungal, and antiviral), antioxidant, antiulcer, and immunostimulant activities [5,6,7,8,9,10,11,12,13]. On metabolic pathophysiology, it has been shown to possess anti-obesity [12], antidiabetic [12], hypocholesterolemic [13], and hepatoprotective [7,12] effects. In the digestive system, it has been shown to maintain [5], ameliorate [6], and regulate [13] gut functions. In the cardiovascular system, it is able to reduce capillary fragility [5], and can improve overall cardiovascular health [6,13]. Some authors reported that BP may contribute to the prevention of and positively impact some degenerative processes such as neurodegeneration [6,14], overall aging [11,13], and cellular apoptosis [15], and may promote recovery from chronic diseases and possess chemo-preventive properties [7]. It has also been shown to improve skin health and reparation, encompassing many valuable cosmetic qualities [5,16].

Due to its established nutritional use, an ISO Norm (Technical Committee: ISO/TC 34/SC 19 Bee products) is being prepared to standardize the quality of bee pollen as a food product (https://www.iso.org/standard/78544.html?browse=tc; accessed on 14 May 2023). This clear interest in BP relies evidently on a solid background of evidence originating from ethnopharmacological heritage and the growingly cumulated experimental data. Although BP in its isolated pellets format may be relatively recent and emerged with the elaboration of mechanical pollen traps, bee bread and plant pollen have been used since ancient times. Ancient Egyptians described pollen as a “life-giving dust” [17], and Greeks believed that pollen and honey gave youth to kings [18]. Pollen was used for cosmetic purposes in ancient China [19]. It has been reported that ancient Egyptians, Greeks, native Americans, Chinese people, and Indians used BP for provisions and energy on long journeys, as well as for other health benefits [20]. Relying on this nutritional and ethnomedicinal history, BP use has been spreading at a very rapid pace during the last few years due to the emerging scientific evidence and the extensive development of the dietary supplement market all over the world. BP is widely commercialized as a stand-alone food and nutritional supplement benefiting from its rich and well-balanced composition. The global market is expected to reach around EUR 670 million next year, 2024 [21].

However, despite the rapid pace of basic and experimental research works on BP, translational and clinical research is still lacking. We have therefore deemed that the extent of currently available evidence may possibly warrant the necessity to step forward and ascertain the translational promises of this evidence. Such promises appear, presumably, to be very encouraging due to the many considerations that we will thoroughly develop during this work. However, reviews that assess such prospects, especially relating to BP’s nutritional but also pharmacological applications, are still lacking.

Hence, the goal of this work is to give an up-to-date review on the evidence-based nutritional values of BP and explore its possible applications in preventing and managing certain nutrition-associated disorders, viz. digestive, metabolic, and cardiovascular disorders, by translating the cumulated experimental and preclinical evidence into clinically relevant insights. We will mainly discuss some chronically evolving disease risk factors such as nutrient deficiencies, oxidative stress, and inflammation, and then focus on pathophysiological conditions that are closely related to nutritional factors such as digestive, metabolic, and cardiovascular diseases.

## 2. Materials and Methods

Due to the lack of interventional studies investigating the clinical use and outcomes of BP in humans, we decided to undertake a scoping review of the currently available evidence supporting the nutritional interest of BP in normal and pathophysiological conditions. We deemed that the current level of evidence and stage of research advancement on BP interest in human health is still in its very early phases, and a literature review is therefore needed to guide future works at all research stages. Scoping reviews are a relatively new approach in evidence synthesis [22,23,24,25]. This review type is especially emerging as a valid methodology in approaching broadly diversified topics that also impose an analysis of a wide range of data and topics [26]. This is perfect for the case of BP composition and applications in human health.

Able to encompass all stages of pharmaceutical, medical, and biomedical research including experimental, preclinical, translational, clinical, and real-world evidence phases, a scoping review was then preferable from our point of view to tackle the nutritional value of BP. This is mainly due to the fact that published works on this product are characterized by very rapid expansion, especially in very recent years (a search on PubMed with “bee pollen” as a keyword returns more than 2200 publications throughout the last century, with more than 1000 publications, strikingly, dated in the last five years). We therefore aimed to rationally assess the extent of the evidence, define current knowledge gaps, and formulate adapted recommendations for future research. Although not mandatory in scoping reviews [27], we will make some critical appraisals throughout this research work when we deem it necessary, especially regarding the clinical relevance of reported or discussed findings.

In spite of the fact that scoping reviews are recent in research practice, diverse well-conceived approaches and standardization attempts have been made to frame them and normalize their conditions, accomplishment, and reporting [22,23,25,27,28,29]. Among the guidelines for such purposes is the guidelines checklist of the “Preferred Reporting Items for Systematic Reviews and Meta-Analysis extension for Scoping Reviews” (PRISMA-ScR), which was formulated according to the guidelines of the “Enhancing the quality and transparency of health research” (EQUATOR) group and published in 2018 [29]. Note that PRISMA guidelines for systematic reviews were also updated and replaced in 2021 by “The PRISMA 2020 Statement” to include “new reporting guidance that reflects advances in methods to identify, select, appraise, and synthesize studies”. Notwithstanding that this update concerned systematic reviews and meta-analyses [30], The JBI Manual for Evidence Synthesis, which remains a pivotal reference in scoping review protocols, proposed many changes (https://jbi-global-wiki.refined.site/space/MANUAL/4688844; accessed on 10 April 2023) to PRISMA-ScR that we will also consider in our current review. 

**Figure 1:**
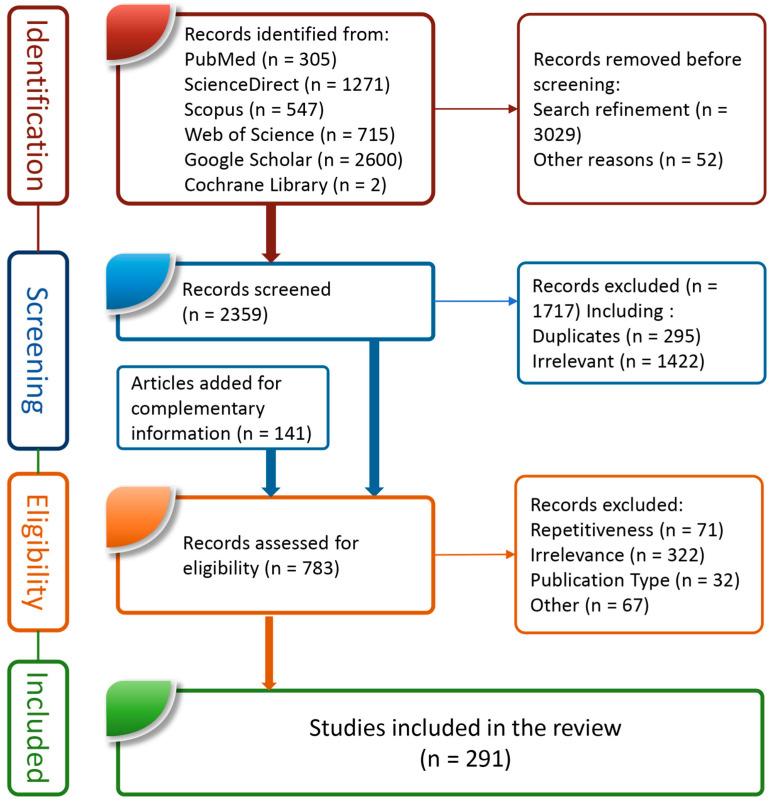
Flow Diagram of Resource Identification and Screening. (The protocol conception relies on PRISMA-ScR and PRISMA 2020 Standards [29]).

As agreed by the JBI manual, changes that were recommended in PRISMA 2020 standards and that are relevant to scoping reviews were considered in items 2, 5, 8–10, 14, and 22 of our PRISMA checklist (see Table 1) when applicable. Our research protocol is summarized in Figure 1 and detailed in Table 1. Moreover, owing to two main considerations, we have focused mainly on publications that were issued during the last four years. Firstly, our preliminary literature research showed repetition in the bioactivity-related parameters that were studied for BP. After analyzing available publications, we have seen that all previously published articles (older than 4 years) reported similar data to that which was available in publications in the last 4 years, evidently relating to our research topic in the current paper. Secondly, two general reviews on BP, dating back to 2020 and before, were published by other colleagues [31,32]. Although no major standardized methodological protocol for literature reviews was claimed or clearly followed by the authors, we have also outlined the main contributions of these two reviews in our current work.

The scientific data for analysis were collected from the main international databases dealing with the medical and pharmaceutical fields, viz. PubMed, ScienceDirect, Scopus, Web of Science, Cochrane Library, and Google Scholar. A preliminary search used the keyword “bee pollen” to screen potentially relevant publications. More affined terms were subsequently used including “bee AND pollen AND nutr*” when the retrieved number of publications was more than 1000 (this was the case with Google Scholar, ScienceDirect, Scopus, and Web of Science). The search term “bee AND pollen AND nutrition” was used in Science Direct because this database does not support searches with wildcard characters (“*” in our case) and have yielded 1271 results. Two filters were then applied: the “article type” filter was limited to “review articles” and “research articles”, and the “subject area” filter was limited to biochemistry, genetics and molecular biology, chemistry, medicine and dentistry, veterinary science and veterinary medicine, pharmacology, toxicology and pharmaceutical science, and immunology and microbiology areas were selected. A total of 305, 417, 547, 715, 2, and 2600 publications were retrieved from PubMed, ScienceDirect, Scopus, Web of Science, Cochrane Library, and Google Scholar, respectively. The search term ““bee pollen” AND nutr* AND doi”, with a 2020–2023 date interval, and just English, French, and Spanish language filters, was used in Google Scholar to refine the search. This refinement resulted in 373 publications after selecting only “review articles”. From the initial 2359 articles that were initially assessed by overviewing the titles and abstracts (when available), we have eliminated the following types from consideration: duplication across databases, books and conference proceedings, irrelevance for our research topic (they were mainly focusing on bee life or, to a much lesser extent, on other bee products, pollen allergy, and BP studies for pharmacological activities that were not considered in this study), and publication language other than the three chosen.

During the time in which we conducted our work, other searches were undertaken to validate the provided data about complementary scientific areas such as human pathophysiology. We have mainly conducted searches for up-to-date reviews of disease mechanisms and the implications for oxidative stress, inflammation, nutrient deficiency, and imbalances in disease onset and outcomes. These pursuits were conducted in PubMed and ScienceDirect. In PubMed, the “review” filter was applied. In both databases, only the last three years were selected as publication dates. We also conducted a search for literature reviews that investigated antioxidant and anti-inflammatory study methods in both databases to guide the structuring of our review of BP activities on these two biological processes.

Finally, 783 full-text articles, including mainly those related to BP, but also some related to honeybee life, human pathophysiology, and research methodology, were fully analyzed and 291 were included in this review. Only those articles published in indexed peer-reviewed journals, having a digital object identifier, and published in English, Spanish, or French, were considered.

## 3. Results and Discussion

### 3.1. Nutritional Value of BP

**Figure 2:**
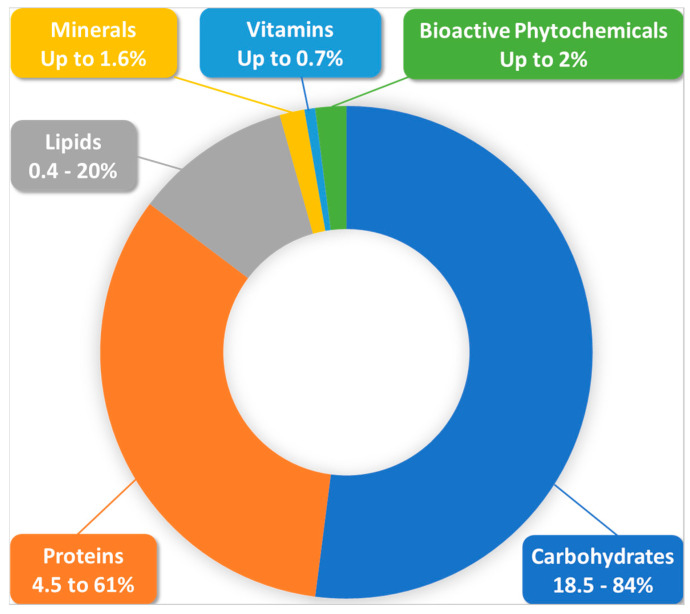
General Composition of Bee Pollen According to the Currently Available Literature.

BP’s richness in nutrients, such as proteins, vitamins, minerals, oligo-elements, and unsaturated fatty acids, is certain, along with the fact that it provides a moderate calorie intake as well as a good level of tolerance and safety, except for in the case of allergic reactions or external pollution, which remain manageable and predictable. BP hazards can arise mainly from external contamination factors, as pollen is very sensitive to these factors [2], or from inconvenient storage and processing conditions [1]. Special requirements are being established in the ISO-TC34-SC19-WG3 norm to avoid such tampering in the quality assurance and control of the crude material. Allergic reactions to the product can be largely prevented if we document pollen composition and preliminarily consider patient sensitivity; BP remains safe for most physiological situations including in childhood, in the elderly and in recovering patients [3,4]. BP can also be a source of many essential elements for breastfeeding and pregnant women. A recent study of 27 different brand commercial BP samples showed that a daily consumption of 40 g of this product by a breastfeeding woman could bring 23.9%, 22.6%, 23.8%, and 26.3% of daily needs of copper, iron, manganese, and selenium, respectively [33]. Similarly, the same study showed that the same schedule consumption by a pregnant woman can supply 31.1%, 30.9%, and 30.7% of copper, manganese, and selenium daily needs, respectively. Moreover, some experimental studies reported that BP had protective effects against prenatal exposure to neurotoxic contaminants in animals [34,35]. Therefore, it appears a priori to be a very good food in basic nutrition, as a complementary functional food, or also as an adjuvant in different pathophysiological contexts. An abridged diagram of BP composition is presented in Figure 2.

According to a recent review of more than 100 published studies, the main components of BP are, in order of weight/weight importance: carbohydrates (percentages from 18.5 to 84% were reported), proteins (4.5–61%), lipids (0.4–20%), fibers, ash, and other components [31,36,37]. Carbohydrates exist here as two types of structurally and functionally distinct pools, namely structural carbohydrates, which contribute to pollen grain structure and protection, and non-structural carbohydrates, which are easily digestible and are generally studied and measured in experimental studies [38]. The high carbohydrate content is mainly due to the blending of plant pollen with nectar by honeybees during pellet formation, but is also modulated by plant species, growth level, and harvesting conditions [31]. BP carbohydrates consist mainly of monosaccharides with fructose and glucose as the main ingredients, and disaccharides such as sucrose, maltose, maltulose, and trehalose [31,39,40]. Polysaccharides are encountered in pellet-covering layers (e.g., sporopollenin in the exine and cellulose and pectin in the intine) where they play an encapsulating and protective role against physical, biological, and chemical degradation, and do not generally have any known nutritional value [31,39,40].

It Is important, moreover, to note that the abundance of polyols (e.g., mannitol, inositol, xylitol, ribitol), as included components of carbohydrates in this matrix, contribute to its lower caloric value while ensuring the equilibrated import of energy sources and other nutritional needs [5].

The second key components of BP are proteins. Some studies reported that protein content may reach 61% in some pollen types [37]. Further to the main botanical origin, the great variation of protein composition may also originate from the fact that BP is mono- or multi-floral [2]. Some comparative analyses reported that pollen was richer in amino acids than eggs, cow meat, and milk [41]. The protein content in this product is frequently regarded as a quality index of its nutritional value [7,42]. Indeed, BP is a natural protein source for honeybees, and thus constitutes a key nutritional supply to ensure the development and growth needs of colony members are met, as well as to serve, via the resulting bee bread [43], as a raw material for royal jelly secretion to feed the larvae and queen [44]. Protein supplementation from pollen may even have some advantages over other widely used protein supplements such as whey proteins. A study in Wistar rats has reported that this supplementation was more efficient as a hepatoprotective compared to whey protein, both in running and non-running animals. This was manifested particularly by enhanced hepatocyte activity and reduced glycogen deposition [45]. Moreover, BP is different from pollen, which is native on the plant. A recent comparative proteomic analysis of bee- and manually-collected pollen of dandelion showed that the bee-collected one contained more metabolism-specific proteins and honeybee proteins which are not found in manually-collected pollen [46]. This study, however, reported that total protein content differed only slightly between the two samples.

Despite its composition variability BP contains all the essential amino acids (AAs) needed by the human body, as well as others used to build proteins in humans [47]. However, this does not mean that all types of pollen contain all these AAs. Some floral sources may lack important AAs that are even essential for bee growth and life. *Taraxacum officinale* pollen, for example, was found to lack tryptophan, phenylalanine, and arginine [46]. A sample of *Helianthus annuus* BP was found to contain phenylalanine at levels that are lower than those needed by honeybees in their normal nutritional requirements [48]. Methionine and valine, which are also essential to honeybees, were found to be absent in BP samples from *Cyanus segetum* and *Cytisus scoparius*, while present in notable amounts in other BP samples from these same species [49]. These differences must be verified by further studies on BP from other species, and those emanating from the natural presence of AAs have to be well distinguished from those originating from experimental procedures. Moreover, in the same geographic location and environmental conditions, BP composition in amino acid could be variable and specific to the individual bee colony [50]. Of course, some of them are more widely present than others. A recent study of BP samples from 32 botanical species showed that arginine, asparagine, glutamine, leucine, lysine, and proline constitute about 60% of the total protein content of BP [49]. We should also underline that the recommended ratios of essential/total AAs are not met in all BP types and that, frequently, some of these molecules are found at limiting levels (qualified, then, as limiting AAs) [7,49]. These levels subsequently limit the total amount of proteins that can be synthesized in the human body following a pollen supplementation, even if total protein intake appears to be sufficient or even high. Therefore, an index called an “Amino Acid Score”, defined as the proportion of the limiting essential AAs in pollen divided by the standard accepted proportions of essential AAs that are considered optimal for protein utilization, was established by some authors. This allows a better assessment of BP protein richness and gives a more accurate estimation of its nutritional value [49].

We should then underline that these data could evidently be of great pharmacological and clinical relevance. The main proteins found in BP are albumins, globulins, glutelins, prolamines, and enzymes [51], but, considering the amino acid-related parameters that we have already discussed, it is the amino acid composition rather than the protein composition that really determines the biological and nutritional value of BP [14]. In fact, hundreds of proteins have been isolated in this crude material. One study of four pollen samples, for example, identified 207 proteins in them [3]. To further enhance BP proteinic value, the enzymatic disruption of pollen walls (by an enzymatic mixture of cellulase: pectinase: xylanase: papain, at 4:2:1:3 ratios) was reported to not only enhance protein liberation and availability from inside pollen grains, but also to permit, unlike physical methods (e.g., ultrasound-assisted and/or freeze-thawing wall breaking), the release of the proteins that form these walls [6].

The third main component of BP are lipids [49,52]. In addition to proteins, lipids appear to play decisive roles in the pollen assembly by honeybees. In fact, it has been shown from different plants and bees species that protein-to-lipid ratios of pollen can drive bee foraging behavior and health [53]. This consideration would be crucial if beekeepers want to “guide” bee foraging and naturally adapt the composition of collected BP for human or animal use. Studies in bumblebees have shown that lipid intake is strongly regulated (more than carbohydrates), and that these insects may overeat proteins to reach a sufficient lipid intake or adequate protein–lipid ratio [54]. Such behavior is not yet studied in honeybees and may, if verified in social honeybees, give important insights about naturally adapting the composition of BP. Bumblebees and honeybees have been shown to possess several similarities in foraging behaviors, and a similarity in lipid and protein foraging is consequently not impossible. Among behavioral similarities, some floral volatiles were shown to attract both insects [55]. A comparative study of honeybee and bumblebee honeys from different geographical and botanical origins reported a similar qualitative composition of free AAs between the two types of honeys, although the quantitative composition was different [56]. Another comparative study reported recently that honeybees and bumblebees both avoid foraging nutritionally poor (e.g., having low protein and high lipid content) and highly toxic pollen, compared to other insects [57].

BP lipids are mainly composed of phospholipids (mostly glycerophospholipids such as phosphatidylcholines, lysophosphatidylcholines, phosphatidylethanolamines, phosphatidylglycerols, and phosphatidylserines), and polyunsaturated fatty acids, but numerous sphingolipids (mainly ceramides), and glycolipids are also present in BP in smaller quantities [58,59,60]. Sterols are also widely present [54], since BP is the only source of these vital molecules for honey bees [61]. Phytosterols, viz. sterols, have also been found to drive phylogenetic signals in bee foraging behaviors [49] and have been linked to bee colony health and performance [53,54]. To our current knowledge, stanols in BP, although frequent in plant pollen [62] and known for their beneficial effects on human health [63], have unfortunately not yet been studied for pharmacological and nutritional purposes.

Long chain fatty acids such as linoleic (Omega-3), α-linolenic (Ω-6), oleic (Ω-9), and α-palmitic acid (saturated) are among the major fatty acids in BP [5,64]. Unsaturated fatty acids may reach 60% of pollen lipids, while palmitic acid is the most abundant saturated fatty acid [65]. Other fatty acids such as oleic acid and myristic acid have also been reported as the main lipidic components [43]. The storage conditions [66], manipulation and processing of BP may alter its lipidic profile. Drying with different techniques, for example, which is the most common operation in pollen use by humans, was shown to reduce its lipid content and alter the structure of its fatty acids, with freeze-drying having the least impact [59]. The lipid profile of BP has also been shown to vary with geographic location and environmental conditions, even for those that are derived from the same plant species [42]. Furthermore, harvesting season was also reported to significantly influence lipid profile. An analysis of pollen loads harvested around one year in the same geographic location showed that, not only did the percentage of different fatty acids in the total lipids vary according to the period of collection, but also that the ratio of unsaturated to total fatty acids was variable during the year with a maximum registered in the summer period [66].

Pollen lipids are not only important for bee life and in assessing BP nutritional profiles. Recent studies showed that these lipids determine the lipidic content and profile of other derivatives produced by honeybees, namely royal jelly [67]. In contrast to proteins, the great impact of the lipidic profile on pollen importance has only just begun to draw attention [68].

BP is also rich in many other nutrients in terms of vitamins and minerals, with a content of up to 0.7% of the total weight being constituted by vitamins, and with being a potential source of considerable amounts of fat soluble vitamins (e.g., pro-vitamin A, vitamins D, and E, which is present in many tocopherol derivatives). It could also contain water soluble vitamins (e.g., vitamins of the B group including B1, B2, B3, B5, B6, B7, B9, and B12, and vitamin C) [5,32,40]. BP is, for example, the richest known source of riboflavin in all plant-based materials [69]. Interestingly, according to numerous comparative studies from different regions of the world [5], the vitamin content of bee bread appears to be richer than BP (including the presence of vitamin K, which is rarely found in pollen). This may likely result from pollen fermentation by lactic acid bacteria from honeybee stomachs and by the bee bread microbiome [40].

Mineral elements are found in BP at an abundant ratio of about 1.6% [33]. They include, at largely variable levels between individual taxonomic types, macro-elements such as calcium, magnesium, phosphorus, potassium, and sodium, and microelements such as copper, iron, manganese, selenium, and zinc [5,32,33].

A large number of other micronutrients includes carotenoids [70], anthocyanins, glucosinolates, and coenzyme Q10, which are well known for their potential nutritional and health-related effects [5]. Numerous enzymes and coenzymes, as well as nucleic acids, particularly ribonucleic acids, are also universally found in considerable amounts [16,32,71]. Betaines were also recently discovered to be widely present in variable quantities depending on the pollen origin, and have been therefore proposed as additional potential markers for origin identification [72]. An analysis was conducted on Spanish BP samples from different botanical origins (e.g., *Brassica napus*, *Vicia sativa*, *Quercus* sp., *Retama* sp., *Papaver* sp., *Rosa* sp., *Teucrium* sp., *Reseda* sp., *Cytisus* sp., *Rubus* sp.,) and the authors verified the presence of betaines in all of them. Some are pharmacologically well-known molecules, such as betaine, betonicine, trigonelline, and choline, being consistently present at concentration ranges of 7–4910, 264–52,834, 12–3628, and 13–723 mg/kg BP dry weight, respectively [72]. These natural and safe molecules, widely known for their protective role against osmotic and oxidative stresses, especially at hepatic and renal levels [73], are still very scarcely studied in BP. Their wide presence, if verified by sufficient studies, may give additional support to the bioactivity of BP in human metabolic organs and related physiological functions and pathological processes.

BP also contains an interesting microbiome comprising mainly *Lactobacillus* strains and other microorganisms such as the *Pseudomonas* genus and yeasts from *Saccharomyces* genus [74,75]. This microbiome evidently originates, at least partly, from the digestive microbiota of the honey bee (bee saliva), and participates in the fermentation of pollen to manufacture bee bread [75]. The exploitation of these strains will contribute to increase the value of BP for further nutritional applications, as we will discuss bellow.

A significant observation to underline is that BP pellets in their natural form are hardly digestible and therefore do not allow the full release of their nutrients due to the high protection of the encapsulation walls of the pollen grains. These shells may decrease the bioavailability of micronutrients by as much as 50% or more [4]. Some studies reported that the grinding of pollen grains and their dissolution in warm water may increase nutrient bioavailability from 10–15% (values for raw grains) to 60–80% [76]. Studies showed that a preliminary disruption of pollen walls might increase nutrient release and bioavailability [6] and thus enhance the nutritional value and benefits for humans. However, be a “double-edged sword” alter pollen composition, and needs further studies and formulation enhancements.

Fermentation is among the most known processes that may enhance pollen’s nutrient availability. It was reported by many studies that it increases composition richness, either considering nutrients such as vitamins, AAs, peptides, and unsaturated fatty acids, or phytochemicals such as phenolic acids, flavonoids, phenolamides, etc. [69,70,77,78,79,80,81]. BP fermentation was found to potentiate bioactivities, such as those that are antioxidant, anti-inflammatory, antibacterial, and antifungal, and to amplify the lowering effects on many metabolic disorder biomarkers, including body mass index, glycemia, cholesterol, and triglyceride levels [70,79,80,81,82]. These kinds of results were obtained by many well-known and widely used fermenting strains, including bacteria such as *Lactococcus lactis*, *Lactobacillus rhamnosus* [82], *Lactobacillus bulgaricus*, and *Lactobacillus kunkeei*, and yeasts such as *Saccharomyces cerevisiae* [77] and *Hanseniaspora uvarum* [80]. Some studies reported that yeast fermentation resulted in a more pronounced enhancement of nutrient composition than lactic bacteria or mixed (bacteria and yeast strains together) fermentations [83]. It has even been reported that fermentation permitted the reduction in allergenicity of BP by significantly reducing its allergen contents and the immunoglobulin-E-binding affinity of these allergens (this was obtained by fermenting *Brassica napus* BP with *Saccharomyces cerevisiae*) [84]. Indeed, bee bread, which is the natural product of the honeybee-ensured lactic fermentation of BP over the course of approximately one week in the honeycomb, is characterized by a partial alteration of the resistant pollen grain wall and a richer and more balanced nutrient composition, as well as an enhanced digestibility, bio-accessibility, and compound bioavailability in the human body [69,79,82]. The results may evidently vary according to the assessed parameters, but the general tendencies that we have enumerated here are generally reported by almost all studies.

### 3.2. BP as a Functional Nutrient Source

#### 3.2.1. Nutritional Deficiencies

It is estimated that more than two billion people suffer from micronutrient deficiency worldwide [33]. Such deficiencies are perceived by the World Health Organization (WHO) and scientists as a global health concern [85,86]. Evidently, the rich composition of BP fosters primarily its use in human nutrition for rebalancing or preventing miscellaneous nutritional deficiencies and pathophysiological conditions. However, the interpretation of experimental analyses warrants a great awareness before drawing any clinically relevant conclusions. Clinical trials that are continuously undertaken worldwide are broadly known to return conflicting results in a large number of dietary supplements. Evidently, miscellaneous bias and/or experimental drifts or shortcomings may lie behind such controversies; however, the lack of translational awareness may be the major “fausse-route” in such conditions, especially among the scientific community. BP may be an interesting option due to its balanced composition and its high safety but more preclinical and clinical investigations are still needed to fully support such claims.

Deficiencies in vital nutrients are widespread in all regions of the world regardless of the socioeconomic conditions. For example, multi-country surveys reported that more than 40% of the European population, 23% of the American population, and 38% of the Canadian population had vitamin D levels below 50nmol/l, which is the minimal normal value agreed upon globally [87]. Many other studies in European countries reported significant deficiencies in many vital nutrients and microelements such as selenium [88,89], and miscellaneous other trace elements and vitamins [90,91,92].

Nutritional deficiencies can be a modifiable risk to prevent subsequent pathological implications, and BP may therefore be a possible solution in most cases of nutritional deficiencies to prevent the outcome of the worsening of many pathophysiological processes. Among the different compounds, the mineral bio-elements, especially trace elements that are drastically lacking in modern lifestyle dietary habits, can be an added value in this product. Other recently highlighted issues, such as fermentation and prior-to-drying measures, may also be harnessed to ameliorate the profit of this matrix either by preventing its quality loss or by enhancing its nutritional qualities.

#### 3.2.2. Antioxidant Potential

Redox regulation is among the most ubiquitously known mechanisms in the human body. Oxidative stress, as concept that reflects the disturbance of redox regulation, is often roughly and unclearly appraised. The translational research experience has shown in countless examples that experimentally and preclinically shown qualities of phytochemicals are not expressed by relevant outcomes in clinical trials [93,94]. However, oxidative stress is well known to contribute to the genesis and development of a wide range of human diseases, including metabolic [95,96] and cardiovascular [97,98] ones.

BP antioxidant activity is one of its most studied properties [99]. This bioeffect, which usually results from a combination of diverse mechanisms [79], is almost universally present in BP as it was always reported by published studies that searched for it, disregarding when and where they were undertaken [9,32,60,74,79,100,101,102,103,104,105]. Moreover, it has marked significance for its anti-inflammatory potential [65,101]. However, this activity, as is the case for overall BP composition, varies widely depending on bee and plant species, geographical location, soil type, timing, processing, and environmental conditions [79,101,106,107] which can make approaching it difficult. Despite that, it is noteworthy that comparative studies have generally reported that multi-floral pollens exhibit more pronounced antioxidant activity than mono-floral pollens [108]. In addition, it has been frequently reported that BP antioxidant activity correlates, sometimes linearly, with its total phenolic compounds [109], with phenolic acids and flavonoids being reportedly the most active [79]. Polyphenols are very well-known antioxidants and thousands of scientific works have verified this activity for these ubiquitous natural compounds. A countless number of them, including many of those encountered in BP, have been conducted either at experimental or clinical levels, and have concerned many types of human diseases [110,111,112,113,114,115,116,117,118,119,120,121,122,123]. The development of these studies being beyond the scope of our current paper, we will not develop these results here. Nevertheless, recent studies that investigated BP’s antioxidant effects are summarized in Table 2. Vitamins, for example, such as C [124,125], D [126,127], and E [128,129], are also well-documented antioxidants that are found in BP. As another example, polysaccharides from *Carthamus tinctorius* BP were repeatedly reported as having important antioxidant activities, such as upregulating antioxidant enzymes activities and lowering circulating levels of oxidative elements in many locations including the serum, liver and brain [130]. Another important observation to underline is that many studies, excluding some rare exceptions [44,109], have reported that bee bread and fermented BP both present more pronounced antioxidant activities than bee pollen alone [69,78,79,82].

**Figure 3:**
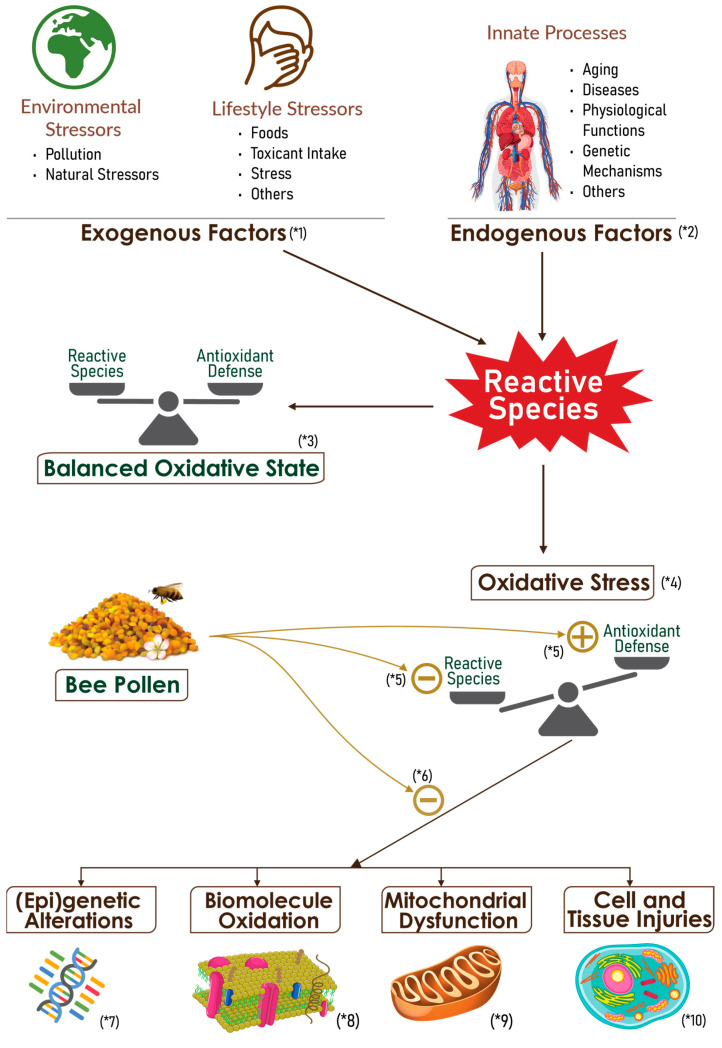
Oxidative Stress Mechanisms and Bee Pollen Action.

Figure 3 explanation:

(*1): Miscellaneous exogenous factors may trigger free radical production in the body

(*2): Human body normally produces free radicals to ensure physiological functions.

(*3): In normal conditions, a balanced state is achieved between reactive species production and antioxidant mechanisms. 

(*4): If the reactive species amount is excessive, or antioxidant mechanisms are insufficient, there is an abnormal accumulation of circulating reactive species and thus an impaired redox balance that is known as oxidative stress.

(*5): Bee pollen can act either by reducing reactive species levels by diverse mechanisms, or by enhancing endogenous antioxidant defense mechanisms.

(*6): BP action results in palliating deleterious oxidative stress consequences that are schematized from (*7) to (*10)

(*7): Oxidative stress results in genetic and epigenetic alterations.

(*8): Reactive species induce and oxidation of biological macromolecules, mainly of lipids and proteins. Lipid peroxidation may dangerously result in altering the structure and functions of plasma membranes.

(*9): Free radicals are also produced by mitochondria for physiological purposes, or as a response to diverse pathophysiological situations or other stressors. 

(*10): The cumulation of diverse oxidative mechanisms and byproducts may result in altering cell functions and structure(s), and ultimately lead to cell death and generalized degenerative processes.

Available evidence, which comes mainly from experimental studies, supports potential multitarget activities for BP that have been shown to encompass at least the aspects illustrated in (*1)–(*10) in this figure (information reported in Table 2).

Oxidative stress can emanate either from an overproduction of reactive species or from an impairment or insufficiency of inner antioxidant mechanisms. A wide array of methods have been used to assess the antioxidant potential of natural compounds by measuring diverse molecular markers such as free radicals, antioxidant enzymes and oxidation byproducts [176,177,178]. In BP studies, all these types of analytical methods have been used but we will give only some illustrative examples from most recent studies. The known mechanisms of BP antioxidant activities are illustrated in Figure 3.

A hydroethanolic extract of a commercial multifloral BP was recently reported to manifest, in a dose-dependent manner, a radical scavenging activity on diverse test-generated radicals (2,2-diphenyl-1-picrylhydrazyl (DPPH) which is classically used in experimental studies, and nitric oxide, and superoxide which were used for the first time in pollen studies) [154]. Water and hydroethanolic extracts of *Cannabis sativa* were also reported to show an in vitro radical scavenging and peroxidase inhibitory activities with hydroethanolic extract being more active [179]. A study of hydroethanolic extracts of many BP samples, including monofloral (*Quercus palustris*, *Actinidia arguta*, *Robinia pseudoacacia*, and *Amygdalus persica*) and multifloral ones, reported that sixteen of the eighteen samples presented a potent DPPH scavenging activity [105]. Another recent study analyzed the antioxidant activity of twenty BP samples by assessing the 2,2′-azino-bis (3-ethylbenzothiazoline-6-sulphonic acid (ABTS)) cation elimination, the cupric ion reducing antioxidant capacity (CUPRAC), and the DPPH free radical scavenging, and reported that all studied samples presented a marked antioxidant potential which evidently varied according to the botanical origin of the BP [180]. Interestingly, this study unveiled two important observations. Firstly, hydrolyzed fractions (the treatment of extractable fraction by methanol–sulfuric acid, 10:1 mixture) of BP were more potent as antioxidants than extractable fractions (hydrochloric acid/methanol/water at 1:80:10, *v*/*v* mixture) in all used assays and samples. This is in accordance with other studies that have shown that enzymatic hydrolysis improved the antioxidant activities of BP [133,181]. Secondly, the measurements showed that the DDPH assay revealed a largely lower antioxidant activity compared to the other two methods (ABTS and CUPRAC). The authors then concluded that the DPPH assay was not suitable to study BP’s antioxidant potential. Since the DPPH assay is among the most used tests to assess the antioxidant potential of natural products and other chemicals [101,182], this intriguing observation should be validated by other BP studies.

The cellular antioxidant activity (CAA) assay is a recently developed method to assess antioxidant activity and is regarded as a better alternative to traditional chemical methods, as it considers the cellular uptake, distribution, and metabolism of the antioxidant compounds [183]. A topical study using this technique in a human hepatic cell line reported that the hydroethanolic extract of rape BP showed significant dose-dependent antioxidant activity that was largely more pronounced with fermented BP compared to unfermented BP [70]. Another study of the ethanolic extracts of different BP samples, including mono-species, -genus and -family botanical origin pollens, and using the CAA assay for the first time in an ex vivo system based on human erythrocytes, reported that all tested extracts induced a significant antioxidant activity in studied cells, with the *Eucalyptus* BP being the most potent and *Erica* BP being the least potent. Other BP samples in this study were *Trifolium pratense* for monofloral BP, *Prunus*, *Rubus*, *Viburnum*, and *Rosa* for BP composed mainly of one botanical genus, and *Brassicaceae* and *Asteraceae* for BP composed mainly of one botanical family.

The antioxidant potential of BP was also assessed by investigating the cellular and biochemical effects of oxidative stress and measuring their variation as a whole product or as an extract. The ethanolic extract of chestnut was also reported to reduce DNA oxidative damage products by 33% [152]. Ethanolic extract of *Schisandra chinensis* BP was shown to significantly upregulate superoxide dismutase activity and glutathione levels, reduce lipid peroxidation product malondialdehyde, and to markedly improve cell morphology and survival rate in a cardiomyocyte model culture [168]. This study also reported that the extract protects remarkably against H_2_O_2_-induced apoptosis by an intricate combination of mechanisms including a significant downregulation of Bax, cytochrome C, and caspase-3 mRNA expression, and an upregulation of Bcl-2 mRNA expression. Accordingly, a comparative study of the ethanolic extracts of *Castanea*, *Cistus*, and *Rubus* BP showed that they significantly reduced diverse molecular markers of oxidation and prevented erythrocytic hemolysis [156].

The antioxidant activity of BP is generally reported either when the study protocol relies on administering raw BP as whole product, or when diverse types of extracts are used. Numerous studies have reported that the feeding of BP to animals resulted in a marked activation of diverse antioxidant mechanisms and an improvement in antioxidant defense against different experimentally-induced oxidative damages [132,137,142,172,184,185]. In addition, studies of BP extracts obtained by diverse solvents, including ethanolic [148,156,167,168], hydroethanolic [15,154,171,179], and diverse organic solvent-mediated extracts [150,151,153,175], have noted important antioxidant activities regardless of the extraction media. These bioeffects were also widely reported in animal models by studies that focused on different anatomical organs and physiological systems including renal [174], nervous [139,174], digestive [144,151], and reproductive [172,185] systems, as well as on different biochemical, hematological, and toxicological markers at systemic levels [137,165,184].

Therefore, BP’s antioxidant potential appears to be very intricate and, surprisingly, encompasses many complementary pathways, ranging from reactive species’ scavenging and antioxidant defense activation to the abolition of the deleterious effects of oxidation byproducts and the resulting protection of cells against lethal injuries such as apoptosis and hemolysis. At the current level of knowledge, these mechanisms remain to be fully elucidate and their clinical relevance remains to be explored. Moreover, it is very important to underline that, during our reviews, we observed that BP samples are frequently supplied by commercial sources rather than by traceable and standardized sampling channels. This is perfectly understandable due to the costly and time-consuming nature of such channels, but, unfortunately, this is very likely to cause avoidable experimental result irregularities and alter the relevance and quality of research works. This observation evidently applies to the study of all BP aspects, either concerning composition, bioactivities, or safety.

#### 3.2.3. Anti-Inflammatory Potential

Chronic low-grade inflammation has been widely recognized as a pathogenesis factor in a wide array of highly detrimental pathophysiological conditions such as auto-immune, cancerous, cardiovascular, metabolic, musculoskeletal, neurodegenerative, psychosomatic, reproductive, dermatological, and other disorders [186,187,188,189,190,191,192,193,194,195]. BP encompasses marked inhibitory activities on inflammations including systemic and localized forms such as neuroinflammation and digestive wall inflammations [65]. The anti-inflammatory potential of BP was amply studied and diverse mechanisms were spotlighted, especially at experimental level [11]. The most recent studies are summarized in Table 3. These activities have been evidently linked to the classically known anti-inflammatory phytochemicals such as polyphenols, but also to other phytonutrients of BP such as lipids, peptides, polysaccharides, and other compounds [11,171,181,196,197]. In addition to the obvious differences according to the botanical origin of BP, differences in anti-inflammatory activity were reported according to bee species. A comparison between the stingless bee, *Melipona fasciculata*, and the European bee, *Apis mellifera*, showed that BP collected by the former showed higher anti-inflammatory and antinociceptive activities [198]. Such observations may be of potential importance in adapting BP as a raw material for variable subsequent uses and indications.

On the common systemic inflammation markers, BP was shown to exert anti-inflammatory effects via diverse mechanisms. Rape BP hydroethanolic extract was reported to exert a potent inhibitory effect on nitric oxide and some proinflammatory cytokine production, and on Cyclooxygenase-2 expression in a macrophage culture [70]. This effect was significantly potentiated after fermenting BP with *Saccharomyces cerevisiae*. A potent and dose-dependent inhibitory activity on both Cyclooxygenase isoforms (COX-1 and COX-2) was also shown for an hydroethanolic extract of pollen (the authors did not mention botanical origin) from a stingless bee (*Scaptotrigona affinis postica*) [171].

Regarding neuroinflammation, a commercial BP feeding was reported to significantly downregulate pro-inflammatory cytokines such as Interleukins 1A and 6 (Il-1A and IL-6) and Interferon gamma (IFN-γ) in autistic animal models [65]. A multifloral pollen was also reported to significantly suppress neuroinflammation by lowering hippocampal levels of the proinflammatory cytokines TNF-α and IL-1β [139].

In the digestive system, a multistep hexane–ethanol extract of *Camellia sinensis* BP was reported to significantly upregulate transforming growth factor beta 1 (TGF-β1) gene expression, downregulate TNF-α and IL-6 gene expressions, and inhibit the mitogen-activated protein kinase (MAPK) signaling pathway in an in vitro inflammatory bowel disease (IBD) mimic [162]. In addition, this study noted that the studied extract reduced gut membrane permeability, reestablished the metabolic profile of tight junction cells, and exhibited other metabolic and antioxidative effects that alleviated the IBD condition and ameliorated the overall intestinal barrier function. In another study, the ethanolic extract of a multifloral pollen composed mainly by *Castanea sativa* (88.8%) and other plants, showed a cell protective effect against TNF-α-induced inflammation in a human colorectal adenocarcinoma cell line. The available mechanisms included the downregulation, in a dose-dependent manner, of many inflammation mediators such as Il-8, COX-2, and the cell surface inflammatory glycoprotein intercellular adhesion molecule 1 (ICAM-1) [11].

The anti-inflammatory potential of BP does not only rely on polyphenols. Protein hydrolysates have been widely researched for their prospects as prominent anti-inflammatory alternatives and are ubiquitous in natural resources [207,208]. BP protein hydrolysates, produced with Alcalase, Flavourzyme, and Neutrase enzymes from commercial BP samples, manifested potent nitric oxide scavenging activity, with IC50 (the concentration of BP hydrolysate that scavenges 50% of the nitric oxide radicals) being largely lower than that of the curcumin used as a positive control [181]. As another example, a water-soluble polysaccharide fraction from *Fagopyrum esculentum* BP manifested a pronounced anti-inflammatory activity by lowering pro-inflammatory cytokines and secretory immunoglobulins A, and acted through gut microbiota modulation to result in many inflammation-countering mechanisms in mice [196].

Many other nutrients which are known to be present in BP, such as vitamins (e.g., A, B group, C, D, E, and K [209,210]) and unsaturated fatty acids [211], are also widely known for their anti-inflammatory effects. In addition to all of those compounds, tannins [171] and glucosinolates [197] have also been shown to exert important anti-inflammatory activities through diverse mechanisms.

**Figure 4:**
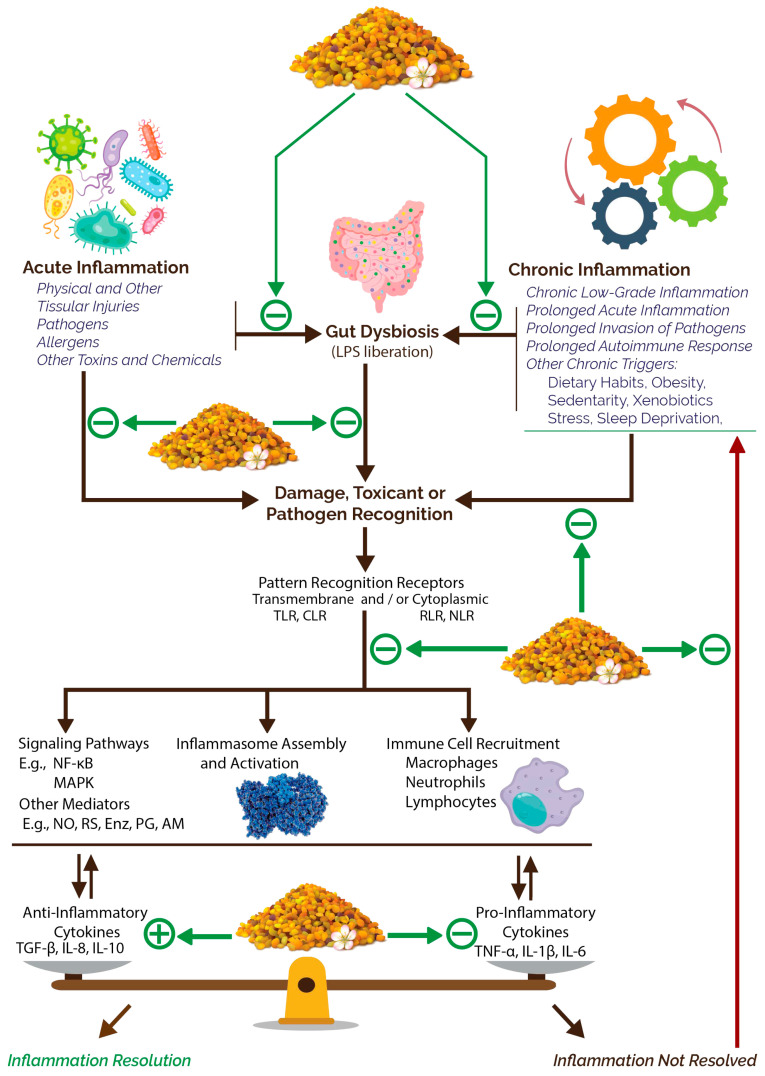
Main Identified Mechanisms of BP Anti-inflammatory Effects.

A brief illustration of the mechanisms that have been identified so far in experimental studies for BP anti-inflammatory effects is presented in Figure 4. It is important to underline that the studies of these parameters are still in their very early phases. Numerous interactions may influence unveiled parameters and outcomes. Striking contradictory results are generally rare in studying the anti-inflammatory potential of BP since this potential has been well established by experimental research. However, many differences in effect extents and mechanism specificities are certainly due to the great variability in BP composition, and numerous studies, as we have seen in Table 3, do not even give precise information about the botanical and geographical origin of the BP samples used. Most studies focus on the total content of phenolic compounds. In the best cases, mostly in total flavonoid compounds and seldom, if ever, contain characterizations of molecular compounds and, extremely rarely, a consideration of BP compounds other than phenolics in studying anti-inflammatory effects. In silico methods which may be of great benefit, especially with emerging omics and computational technologies, have also only recently been used in some studies. Moreover, studied mediators in experimental studies may not always be reliable in correctly assessing anti-inflammatory effects. The role of some examples that we have seen in this section may give a clear insight about this problem. IFN-γ, for example, is a pleiotropic mediator that may present anti- and pro-inflammatory roles depending on the pathophysiological contexts and triggering mechanisms, and its role is still not well understood [212,213]. The mechanism of pleiotropy is a frequent trait in cytokines that still hinders the understanding of their mechanisms and makes it difficult to clearly and firmly establish their roles in different pathophysiological situations [214]. The distinctive roles of the two macrophage subtypes on inflammation has been also shown by some studies [215] to not be as simple as they are classically appraised (M1 are classically considered as pro-inflammatory and M2 as anti-inflammatory). Future studies must therefore consider the knowledge gaps in pathophysiological mechanisms, and adopt a more all-inclusive approach in choosing the parameters to study and those to consider in preparing the raw material to use in experiments.

Another extremely important consideration that is still omitted in most studies lies in the fact that numerous pathologies that are among the most burdensome in our current world come from chronic inflammation, and most studies that have been undertaken so far evaluated the anti-inflammatory effect of BP in rapidly-produced experimental conditions which may not reflect the real pathogenesis evolution and the real BP effect in simulated disease models. Most of the diseases in which inflammatory processes are involved progress over many years or decades and, thus, any evaluation of possible corrective means must consider such a fact.

Furthermore, since the anti-inflammatory effect of BP may emanate also from a large array of its constituents, it is crucial to consider that the effect of them on the treated patients or the at-risk persons that may take BP for preventive purposes will not be the same as in healthy persons. It has been recently shown through many examples that the effects, including the anti-inflammatory ones, of nutrients are altered in inflammatory terrains [216]. A person with an inflamed internal milieu will not benefit in the same way from an anti-inflammatory diet as another person would. These kind of inferences being still recent and not completely understood, further investigations are necessary, especially when considering the differences between the preventive and curative potential of BP as an anti-inflammatory arsenal.

This biopotential is, however, an acknowledged characteristic of this product. Since inflammation is a major mechanism in numerous devastating diseases, especially those linked to modern lifestyle including digestive, metabolic, cardiovascular, and neurodegenerative ones, BP may boast a relevant interest in preventing and managing these conditions. The fact that it exerts its anti-inflammatory effects through a rich mechanism diversity, potentiated by diversified antioxidant mechanisms, remains, however, to be supported by more preclinical and clinical studies.

BP, with a controlled and standardized quality, would be an affordable and safe part of an integrative and balanced dietary intervention to prevent and scale down many pathophysiological conditions, which are weighing heavily on public health in our current societies, without efficient solutions. Innovative means must be ascertained in order to standardize reproducible BP compositions, and give sufficient data, as well as translational research, should be gathered and made available to the scientific community and healthcare practitioners.

#### 3.2.4. Digestive Health

Numerous studies showed that BP may improve gastrointestinal functions in many ways including digestion, absorption, secretions, and microbiota modulation. It encompasses marked gut-protective potential, as was reported by a series of in vitro and in vivo studies. In a human intestinal cell line, a lipidic extract of *Camellia sinensis* BP was found to inhibit dextran-sulfate-sodium-induced cell viability loss, oxidative damage, and epithelial barrier permeability, and to significantly enhance transepithelial and cellular absorption and metabolism of lipids [58]. Using a lipidomic approach, the authors of this study unveiled the contribution to such effects by numerous lipids including those classically known to enhance cellular structure and intestinal barrier components such as monolayer and tight junctions.

The enhancing effects of BP on digestion-related functions in the digestive tract have also been reported by in vivo studies. A very recent meta-analysis reported that BP supplementation to rabbits resulted in an increased activity of digestive enzymes (protease, amylase, and lipase) in the intestinal content, and in an enhanced digestibility of crude proteins, crude fibers, and other organic matter [217].

Intestinal absorption and secretory function may also be enhanced by this matrix. The supplementation of *Brassica napus* BP to rats was reported to significantly increase intestinal crypts depth at 0.2% and 0.5% *w/w* feed ratios, and to decrease it at 0.75% *w/w*, but all ratios led to a significant increase in intestinal villi length [32]. Similar findings were reported in broilers where 1.5% supplementation resulted in an increased density and depth of intestinal crypts, as well as longer and thickener duodenum, jejunum, and ileum villi [32]. Such effects may therefore suggest the potential of BP to improve intestinal mucosa through enhancing absorption and secretory functions.

Many in vivo studies reported that BP modulated digestive tract microbiota in a favorable way. Many examples of digestive function ameliorations and microbiota enhancements or dysbiosis corrections were already given in Table 3, as microbiota are currently very widely known to modulate the inflammatory response in the digestive tract, but also in many distant localizations of the body. Some recent examples are given below.

In the oral cavity, BP fed at a ratio of 5% to rats for one month resulted in a marked increase in the genus Lactococcus (*Lactococcus lactis* is known to prevent oral pathogen spread such as *Streptococcus* mutans and *Porphyromonas gingivalis*) in oral and intestinal flora [218]. This effect in the oral cavity may not be totally due to the antimicrobial activity of BP, because the authors demonstrated that the latter did not have significant effect on *Streptococcus* mutans although a potent inhibitory effect was observed on *Porphyromonas gingivalis,* while an upregulated antimicrobial peptide production by oral epithelium was also noted.

An in vitro study of the hydroethanolic extracts of nineteen diverse pollen samples (*Actinidia arguta*, *Amygdalus persica*, *Quercus palustris*, and *Robinia pseudoacacia* being mono-floral and the others, multifloral) reported that all these extracts did not inhibit human intestinal beneficial bacteria [105]. The tested strains were some of those most widely utilized as probiotics in dietary supplements, viz., *Bifidobacterium bifidum*, *B. breve*, *B. infantis*, *B. longum*, *Lactobacillus acidophilus*, and *L. casei*, and an acidulating bacterium (*Clostridium butyricum*). Meanwhile, the extracts showed weak inhibitory activity on two nonpathogenic bacteria (*Bacteroides fragilis* and *Escherichia coli*) and on many tested pathogen bacteria (*Clostridium difficile*, *C. paraputrificum*, *C. perfringens*, and *Staphylococcus aureus*).

Accordingly, another study in rodents with high-fat-induced metabolic syndrome reported that supplementation of *Brassica campestris* BP sharply reduced proteobacteria abundance, restored the firmicutes–bacteroidetes ratio (which was altered by the high-fat diet), and increased *Lactobacillus* and *Lactococcus* abundance in the gut microbiota [81]. The study also showed that pollen submitted to a wall breaking by yeast fermentation was more effective than normal BP. Likewise, in hyperuricemic rats, *Camellia japonica* BP extract resulted in a normalization of the firmicutes–bacteroidetes ratio, and bacteroidetes, proteobacteria, and *Clostridium* levels [151]. This study also reported a significant increase in *Lactobacillus* and *Clostridiaceae* abundance, and in short chain fatty acids.

It can be inferred that numerous aspects of preventive and corrective bioactivities, in the digestive system structure and function, are verified with BP supplementation or extract administration. This, interestingly, covers nearly all major steps in the course of food digestion. We have seen some intriguing and valuable qualities on gut microbiota such as concomitantly enhancing the beneficial strains amount and microbiota diversity while inhibiting pathogen strains. Unfortunately, studies in humans are still lacking in all of the aspects discussed above.

#### 3.2.5. Metabolic Disorders

BP has proven to improve many metabolic imbalances through different and combined mechanisms, some of them connected to disorders such as obesity, diabetes, and dyslipidemia.

Many studies showed that BP reduces liver fat deposition and lipid levels. In a murine hepatic cell line, the ethanolic extracts of many multifloral pollens manifested significant hepatoprotective effects against experimentally induced free radicals, and efficiently reduced lipid accumulation in liver cells [159]. This steatosis reduction showed a low correlation with total phenolic and flavonoid content, but correlated moderately with quercetin levels in pollen samples. In rodent hepatocytes it increased nucleus size, reduced total and low-density lipoprotein cholesterol (LDL-C), TG, and glycogen deposition, concomitantly with lowering the blood pressure of the portal vein [45]. In rodents, BP reduced the steatosis and liver cell degeneration resulting from a high-fat diet, and prevented nonalcoholic fatty liver disease establishment in obese mice [32,159]. In rats intoxicated by propionic acid, the ethanolic extract of a commercial BP sample was also reported to restore hepatocyte function through normalizing numerous hepatic enzymes and molecular markers of liver injury, mitigating oxidative stress [144]. In rodents suffering Fluvastatin-induced hepatitis, a supplementation of a multifloral BP was reported to reduce many biochemical and histological markers of hepatic injury including circulating enzymes marking hepatic injuries, albumin, bilirubin, liver oxidative stress markers, portal vein congestion, abundance of inflammatory cells, and hepatocyte necroptosis [158].

##### Obesity

Although obesity is classically perceived as a disease resulting from an imbalance between calorie intake and expenditure, this disease has been recently acknowledged as evolving upon a complex interplay of biological and psychosocial factors, and the body has been found to encompass an ambiguous defense mechanism against weight loss even with hypocaloric diets [219]. However, it is at least well known that some biological aspects which are largely discussed in this review are tightly involved in the biological processes of obesity pathophysiology. Gut microbiota have been shown, for example, to be closely related to diverse macronutrient metabolisms and fat depositions, as well as to a large plethora of hormonal and other signaling pathways, making the dysbiosis of the micro-organism community involved in obesity inducement and worsening [220,221]. For instance, intestinal permeability, inflammatory processes, oxidative stress, epigenetic regulation, genetic predisposition, and, of course, imbalanced diet are certainly linked to obesity pathogenesis [220,222,223]. Notwithstanding genetic and lifestyle habits, we have seen, during the previous sections, that BP presented important modulatory effects in most of these pathophysiological factors.

Some in vivo studies reported that BP extracts may result in a marked reduction in body weight (18.23% and 19.37% reduction by 7.86 and 15.72 g/kg body weight of pollen extract *Schisandra chinensis*, respectively) [32], in addition to many effects related to other obesity traits, such as reducing liver fat deposition and its oxidative damage, improving lipidic profile, and enhancing hepatic metabolism and autophagy.

Many of the active phytochemicals in BP are widely studied or known for their obesity-mitigating effects. Flavonoids may be the most illustrative example, among them being flavonols (e.g., kaempferol, myricetin, quercetin), flavones (e.g., luteolin, vitexin), and flavanones (e.g., hesperidin, naringenin). These compounds entail numerous effects on pathophysiological markers of obesity such as lipid peroxidation and deposit inhibition, blood-glucose-lowering, anti-inflammatory, and antioxidant effects, hepatocytes and other protections, insulin resistance suppression, energetic catabolism activation, etc. The same observation may be carried out for other components such as phenolic acids too. These effects differ from one molecule to another, but numerous good reviews have been carried out on this topic and present promising prospects that we could not detail here [111,116,224,225,226,227,228,229,230].

As we have already highlighted, for some disorders, BP appears to have a very profitable potential in at-risk persons as a prophylactic product where it may act more profitably in inhibiting etiological pathophysiological events. It preserves, however, all these benefits in confirmed obesity stages since numerous management protocols of this disorder rely on dietary and lifestyle-changing measures such as meal replacements and low-calorie-based micronutrient supply, and it could perfectly fill these needs at least partly. A substantial amount of studies, especially at experimental and preclinical levels, have been published so far. However, these studies do not generally deal with the complete parameters that are gathered by a clinical picture of the obesity disease situation. Regrettably, studies in humans are still absent. Even in experimental studies, it is evidently noted that experimental protocols frequently lack an integrative approach in their study design, especially regarding variability considerations, investigated parameters, raw material standardization, etc.

##### Diabetes Mellitus

The beneficiary effects of BP on liver function and structure invoke a possible role in improving insulin resistance. This was, for example, recently verified with a polysaccharide fraction isolated from *Rosa rugosa* BP, which lowered glycemia levels in type 2 diabetic mice by reducing hepatic steatosis and insulin resistance [231]. To explore a possible effect in type 1 diabetes, this fraction, tested in vitro, resulted in increased pancreatic β-cell proliferation and insulin secretion, while, in mice, it reduced alloxan-induced fasting hyperglycemia, as well as water consumption, urinary volume, and ketone bodies levels, and markedly prevented β-cell loss, restored β-cell/total pancreas volume ratio, and increased insulin levels [231].

In a human colon cell line, *Camellia sinensis* BP extract significantly reduced glucose transport in the epithelium monolayer at different concentrations and time intervals, and drastically reduced glucose absorption by suppressing the expression of glucose transporter 2 and sodium-dependent glucose transporter 1 proteins [232]. Using molecular docking, this effect seemed to be largely due to phenolic compounds.

The possible antidiabetic action of BP in the intestinal tract was also shown by an in vitro study of a multifloral sample composed mainly of *Echium plantagineum*, *Cistus ladanifer*, and *Quercus* sp. A hydroethanolic extract of this BP resulted in a significant dose-dependent inhibition of α-glucosidase activity which was, however, much weaker than acarbose in all tested doses [154].

In mice models where type 2 diabetes was induced by a high-fat diet and streptozotocin administration, the ethanolic extract of *Fagopyrum esculentum* BP produced a marked decrease in fasting blood glucose and “homeostatic model assessment for insulin resistance index”, and an increase in insulin levels, “homeostasis model assessment-β index” (effect extents were more than metformin at certain doses) [201]. Moreover, this study noted that mice treated with this extract had a significant decrease in triglyceride, LDL-C, alanine transaminase, aspartate transaminase, and the lipid peroxidation byproduct malondialdehyde, an increase in high-density lipoprotein cholesterol (HDL-C), and a marked and dose-dependent restoration of adipocyte enlargement which is induced in diabetic mice.

However, this study reported some intriguing observations. Using two doses of BP extracts (1 and 6 g/kg bodyweight) and metformin in all assays, the authors reported that only the high dose of BP extract effectively reduced the total cholesterol, and a significant increase in hepatic glycogen level was only noticed with the low dose of extract. As pro-inflammatory cytokines are known to be upregulated in diabetic patients, and some of them such as IL-6 and TNF-α may be involved in inducing pancreatic β-cells apoptosis, this study curiously reported that mRNA expression of TNF-α and TGF-β were downregulated only by the low dose of pollen extract while the high dose only downregulated TGF-β mRNA expression but not TNF-α. Moreover, a down-regulated mRNA expression of IL-6 was observed in low-dose-treated mice but not in high-dose- and metformin-treated mice. This study being very recent, more work is needed to elucidate the reproducibility and the underlying mechanisms of these observations. Both BP extract dosages activated the phosphoinositide 3-kinase/protein kinase B (PI3K/AKT) signaling pathway which, albeit involved in a multitude of physiological and pathological processes, promotes insulin secretion from pancreatic β cells and is impaired in type 2 diabetes. As another important effect, both BP extract dosages induced an enhancement in gut microbiota diversity and restored strains that were tested in this study to a profile that is close to normal diet mice.

It is worth underlining that the examples of BP that we enumerated in this section are all derived from well-known plants that have evidence-based antidiabetic effects. Indeed, *Camellia sinensis* [233,234] and *Fagopyrum esculentum* [235,236] were largely reported to exert antidiabetic, anti-dyslipidemia, and protective cardiovascular effects in animals and humans. *Echium plantagineum* oil [237] and *Quercus* sp. fruits [238] have been shown to possess diverse antidiabetic and metabolic-syndrome-lowering activities by human clinical studies. *Rosa rugosa* [239,240] has also been reported, experimentally and ethnopharmacologically, to have such effects. Such observations may be of great interest in guiding bioprospection research, although studies comparing the phytocompound contents of BP, plant pollen, and other plant parts remain very sparse and insufficient. Likewise, clinical trials on BP usefulness in diabetic patients and in early insulin resistance stages are unfortunately still lacking despite the substantial amount of preclinical evidence that is available and the affordability and known high safety of the product.

**Figure 5:**
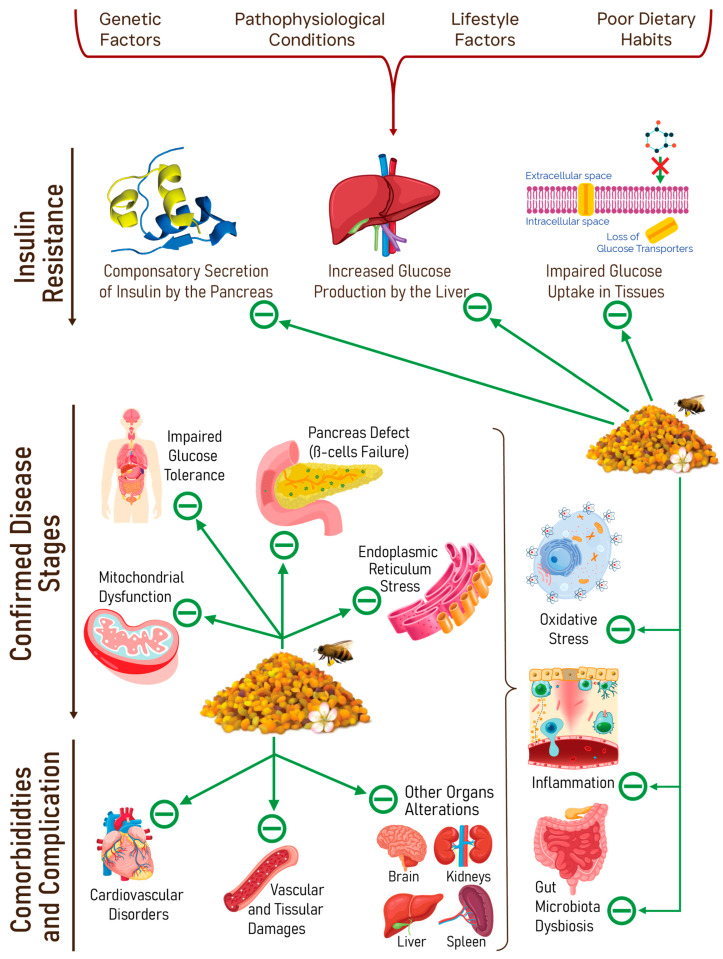
General Mechanisms of BP Antidiabetic Potential.

Studies that have investigated BP bioactivities on pathophysiological processes that may be related to diabetes are too manifold to all be cited here. Indeed, as we are focusing just on recent findings and inferences in this paper, we have exposed only some relevant examples. This allows us to draw evidence-based insights about the promising and multitargeting potential of BP in this pandemic disorder which is, for the most part, settled through a long course of imbalances and is complexly implicated in some of the greatest morbidities of human beings. BP has direct effects on key diabetes biomarkers such as glycemia levels, insulin secretion and resistance, hepatic glycogen levels, enzyme activities and steatosis, β-cells function, fitness and apoptosis, glucose transport, carbohydrate digesting enzymes, and ketone production. A summary of BP antidiabetic potential is illustrated in Figure 5.

A countless number of studies unveiling the antidiabetic properties, including early preventive, disease course modifying, and complication limiting effects, of individual nutrients and phytochemicals that are ubiquitously present in BP from different botanical and geographical origins have been published so far. This includes, for example, nutrients including vitamins such as groups B [241,242,243], C [244,245,246], D [247,248,249], and E [128,250,251], oligo-elements and other minerals [252,253,254], and lipids such as unsaturated fatty acids [255,256,257] and phytosterols [63,258,259], as well as other phytochemicals including polyphenols such as phenolic acids, flavonoids, and stilbenes (very recent and comprehensive reviews of preclinical and clinical evidence can be found in [260,261,262,263,264,265,266]). Despite the substantial amount of experimental and clinical evidence of all these BP compounds in the complex pathophysiology of diabetes and in its interaction with other comorbidities, the possible synergistic potential and real clinical outcome of these compounds, which are always combined in BP, have generally been omitted in research and must be further studied by in vivo experiments, and subsequently verified and objectively assessed by clinical studies. Notwithstanding the biological and chemical diversity of BP, these kind of studies in humans may be more easily reachable than individual phytochemicals and other drugs due to the affordability, wide human use, and potential safety of BP.

##### Dyslipidemia

In this text, it was already seen through many examples that numerous mono- and multifloral BP samples produced marked improvements in lipid profiles in vivo.

As we have already cited, *Brassica campestris* BP supplementation to high-fat-fed mice resulted in a significant decrease in total cholesterol, triglycerides, and LDL-C, but unexpectedly did not result in a significant variation in HDL-C [81]. Another example that we have already given involves a polysaccharide from *Fagopyrum esculentum* BP which induced a significant decrease in triglyceride levels [196]. This fraction also did not significantly affect the HDL-C levels. The ethyl acetate and *n*-butanol extracts of a commercial BP showed an important decrease in the total LDL-C and triglycerides, and a significant increase in HDL-C in rodents, but such effects disappeared when animals were intoxicated with propionic acid [144]. Lipid lowering effects and lipid peroxidation prevention were reported for many monofloral, multifloral, and commercial BP extracts [16,32,45]. In contrast, supplementation in aged horses did not result in such significant changes after 30 days of 60 g/day of pollen supplementation [137].

A daily supplementation of 40 g of pollen in patients with heart failure reduced their total cholesterol levels [60]. In patients with atherogenic dyslipidemia, a mixture of BP, bee bread, and honey induced a marked decrease in total cholesterol (−18.3%), but it is worth noting that bee bread alone reduced total cholesterol by 15.7% [267]. BP alone resulted, in patients with a body mass index above 25, in an improved blood lipid profile after weight loss [267].

Despite seemingly controversial results in animals, which may obviously emanate from different extraction medias and pollen contents, in vitro, in vivo, and human experiments show very encouraging results to pursue research investigations about BP use in dyslipidemia. However, more studies linking BP composition to its lipid lowering effects are needed to have robust data that could provide the evidence for a rebalancing of the diet plans made with this product.

Evidently, lipid profile improvement by BP will be coupled with many other benefits that we have seen previously, such as antioxidative and anti-inflammatory effects, insulin resistance and hyperglycemia alleviation, unsaturated fatty acid, phytosterols, and other nutrient supplies, gut microbiota improvement, and atherosclerosis-risk-lowering potential. This may therefore give BP a general potential in managing dyslipidemias and particularly in preventing their long-term detriments, but such claims must always be verified by well-designed interventional clinical studies.

##### Hyperuricemia

Hyperuricemia is no longer a “disease of kings”, but a widespread disorder which is frequently seen in patients with metabolic syndrome, is tightly linked to some pathophysiological processes such as oxidative stress, inflammation, and apoptosis, and is a confirmed risk factor of disorders such as non-alcoholic fatty liver disease, chronic kidney disease, and cardiovascular diseases [268]. Due to its anti-inflammatory, antioxidant, and apoptosis inhibitory effects, BP may be presumed as a useful product in hyperuricemia.

Studies regarding BP in hyperuricemia models are practically absent. One study that was very recently published reported that the phenolic-concentrated extract of *Camellia japonica* BP markedly decreased uric acid levels in rodents with a potassium-oxonate-induced hyperuricemia [151]. Animals receiving 4 g/kg body weight of the extract manifested restored uric acid levels that were close to those reached by allopurinol (uric acid levels decreased by 72.83% and 75.38% in animals receiving 4 g/kg of BP extract and 5 mg/kg of allopurinol, respectively).

This study invites more detailed investigations of BP in metabolic troubles. It gives an example of how BP, in addition to its nutritional value, may bear other active compounds that may correct, or at least alleviate, nutritional and metabolic troubles.

#### 3.2.6. Cardiovascular Diseases

Cardiovascular diseases, as we highlighted before, can be caused by few or many of the unbalanced situations described above, such as nutrient shortages or imbalances, oxidative stress, inflammation, digestive troubles, and especially metabolic disorders. The main reason why we decided to include this point here is to discuss the possibilities that involve some of the renowned potential benefits of BP to prevent and/or manage cardiovascular disorders.

BP has been shown to exert many bioactivities related to the functions and diseases of the cardiovascular system. In addition to its high anti-inflammatory and antioxidant potential, BP has been experimentally reported to reduce blood pressure [100,269] and circulating cholesterol and triglyceride levels in some animals [137]. Elevated blood pressure and hyperlipidemia are known among the major risk factors for fatal outcomes of cardiovascular diseases, and their reductions are very widely confirmed as cardiovascular risk-lowering measures [270]. Inflammation [193] and oxidation [271] are also among the most marked secondary and aggravating processes in the pathophysiology of cardiovascular diseases. Other troubles such as diabetes, obesity, and other metabolic disorders are also among major risk factors of cardiovascular defects [272,273,274]. Moreover, BP manifested a potential to reduce the secondary cardiovascular complications associated with these metabolic disorders [16].

BP extracts have been shown to reduce hypertension by different ways, emanating mainly from its antioxidant and anti-inflammatory mechanisms, that lead to a marked inhibition of the angiotensin-converting enzyme and general promotion of endothelial functions by modulating the renin–angiotensin system. Such mechanisms were shown to be driven, at least, by polyphenolic compounds [100].

Polysaccharides from *Carthamus tinctorius* BP were also shown to exert a marked anticoagulant effect which was, interestingly, for certain extract fractions, more pronounced than heparin in prolonging prothrombin time in vitro and in human plasma [130]. Cardiac glycosides have also been isolated in hydroethanolic extracts from pollens of *Micromeria fruticose*, *Achillea fragrantissima*, and *Phoenix dactylifera*, but the corresponding BP of these botanical species was unfortunately not included in the study [275]. To our current knowledge, no study has until now focused on the presence and variability of such compounds in this product and this may constitute an interesting research pathway.

Among its possible mechanisms, it induces hepatic lipid metabolism [276], but other systemic effects related to rich pollen composition, such as the elevated ratio of unsaturated/saturated fatty acids [5], and the abundance of phenolic compounds, phospholipids, and phytosterols [277], are also involved. It is worth noting that it was, curiously, supposed that foraging honeybees prefer pollens that contain high levels of unsaturated fatty acids [31].

The anti-atherosclerotic effect was reported by studies in animals and humans for BP and for numerous individual phytochemicals that are mostly present in it, such as some polyphenols. This effect was frequently reported concomitantly with other cardioprotective effects such as stimulating microcirculation, blood clotting reduction, endothelial function and renin-angiotensin-aldosterone system enhancement, and lipid lowering, in addition to the classically reported antioxidant and anti-inflammatory effects [16,32,267,276]. In animal models, the anti-atherosclerotic effect of BP ethanolic extracts was verified through diverse markers, including a marked decrease in atherosclerosis biochemical markers, such as total cholesterol, oxidized low-density lipoprotein, asymmetric di-methylarginine, angiotensin-converting factor, and angiotensin-converting enzyme, as well as an overall reduction in atherosclerotic plaques [16,32,100]. Accordingly, whole BP supplementation in rabbits resulted in a significant decrease in cholesterol and total lipids, as well as in other biochemical parameters that are closely related to atherosclerotic and myocardial infarction risk, such as lipid peroxidation byproduct malondialdehyde, and the infarct marker aspartate aminotransferases [217].

The use of *Castanea sativa* BP was found to efficiently reduce endoplasmic reticulum stress (ER) in human microvascular endothelial cells, an effect which manifested in many ways such as enhancing cell viability, reducing reactive species genesis, and modulating the expression of the diverse factors involved in ER stress [156]. This stress is known to interfere very intricately with an extensive plethora of metabolic and pathophysiological processes, especially in metabolic, cardiovascular, and degenerative diseases [278,279,280,281]. The anti-inflammatory and antioxidative potential of BP may obviously endorse its action on ER stress, but further mechanisms should be investigated as studies that focus on the topic are still very scarce. In the same context, carbonized *Typhae* spp. pollen was found to inhibit endoplasmic-reticulum-stress-induced apoptosis in human aortic-vascular smooth muscle cells, and to additionally exert a significant antithrombotic effect [282]. These results also invoke the importance of the prospect of the different modifications of BP in ameliorating its bioactivities and making pollen production and clinical use more profitable.

In a pouch-induced inflammation model, BP methanolic extract exerted an anti-angiogenic effect in addition to an anti-inflammatory effect, manifested mainly by markedly reducing pro-inflammatory cytokine production [203]. This potent anti-angiogenic effect, although proposed by the authors to be due to TNF-α and vascular endothelial growth factor inhibition, needs further investigation for other possible mechanisms and interferences, as may possibly be the case in cardiovascular and neoplastic diseases.

BP from many plants such as *Brassica napus*, *Nelumbo nucifera*, and *Schisandra chinensis* were found to mitigate induced cardiomyocyte hypertrophy in animal models via antioxidant, anti-inflammatory, and anti-apoptotic mechanisms [15]. *Nelumbo nucifera* pollen was found to significantly reduce cellular damage, prevent cell surface area increase, and suppress pathological protein synthesis [15]. It is worth noting that highly different concentrations of BP extract in this study did not show parallel variability in all three effects (100, 250, and 500 µg/mL were used). In the same context, it was found that BP could reduce the production of the aging promoter lipofuscin in many tissues including cardiac muscle [283]. *Schisandra chinensis* BP showed also a potent and dose-dependent inhibitory effect on the apoptosis of cultured rat cardiomyocytes [168].

The cardioprotective effects of BP, especially toward myocardial infarction, were verified via diverse mechanisms in rodents. In isoprenaline-treated animals, *Schisandra chinensis* BP extract exerted some vital cardioprotective actions in the heart through upregulating, in a dose-dependent manner, nuclear factor-erythroid 2-related factor 2 (Nrf2), heme oxygenase-1, and B-cell lymphoma 2 (Bcl-2) protein expressions, and decreasing Bcl2-associated X protein expression [267].

In experimentally induced hypertensive rats, the administration of BP was found to reduce many tissular injuries in reproductive organs and to prevent the loss of spermatogenic cells. The highlighted mechanisms in this study were mainly the local amelioration of antioxidative defense in testis and epididymis through significantly upregulating important enzymes such as nitric oxide synthase, catalase, and paraoxonase, and through the suppression of inflammatory events by inhibiting the nuclear factor kappa-light-chain-enhancer of the activated B cells signaling pathway [152]. BP could pave the way for drug discovery in cardiovascular medicine, especially in the search for the preventative treatments widely desired by scientific and medical specialists.

A few interesting clinical studies evaluated the effects of BP combinations on the parameters related to metabolic and cardiovascular diseases in humans and generally reported encouraging results including disease-modifying effects in patients with atherogenic dyslipidemia (reviewed in [16,284]), but the pharmacological and clinical relevance of these reports remains to be investigated, since comparative studies or clinical studies of BP alone are still practically absent.

Preventive measures are among the most prominent policies that are widely coveted worldwide to reduce the burden of various illness such as, for instance, cardiovascular diseases that are still unfortunately the leading cause of deaths globally. Sex-specific differences are widely recognized in cardiovascular risk due to numerous physiological differences between men and women [285]. As BP is a promising preventive product, such consideration must be investigated by scientific research, and a possible role and adaptation of BP may be important in this context. To our knowledge, there is no published study on such a topic.

### 3.3. BP as a Food Adjuvant

Many assays are being performed to develop BP’s use as a functional food as well as an adjuvant in food preparations for different purposes. In its natural form, BP is reputed to be a barely digestible product due to the well-sheltered structure of its grains, which hinders the bioavailability of its nutrients and phenolic compounds at high extents that can exceed 90%, and also for some nutrients and other compounds, with a very important variation depending on the floral origin and assessed molecules [99,286,287]. Many safe processing schemes, including fermentation [70,78,80,81], wall-breaking biotechnological, physical and chemical techniques [288], and some guided extraction protocols [2,101], were reported to significantly enhance bio-accessibility to its compounds.

In addition, this product is already widely used as an adjuvant in numerous food recipes to enhance their nutritional values, benefiting from its diverse additive (e.g., antioxidant and preservative) qualities. It is used or studied in many fermented products such as bakery and dairy products, confectionary, meats (e.g., livestock and broiler feeding) and meat derivatives (e.g., frankfurter sausages), and fruit juices [4,8,31,44,140,289], or to change the nutritional and bioactive values of other bee products such as honey [51].

BP also possesses many functional properties that may serve to enhance textural and other formulation aspects in foods. It has, for example, a high emulsifying potential, oil retention capacity, and carbohydrate solubility, but may also have a significant water holding capacity and foaming properties. All these properties are very closely related to pollen composition, except some that are still not fully understood (e.g., water and oil holding capacity was found to not correlate with protein, carbohydrate, or lipid contents, nor with other functional properties) [31]. This kind of observation provides another argument that BP composition and properties are just beginning to be understood.

## 4. Conclusions

This is the first comprehensive review on the nutritional aspects of BP that has followed a structured and standardized approach. The scoping review, which analyzed all works that have been published in the last four years, summarized and unveiled the evidence for human nutrition, especially in at-risk and other vulnerable persons. The antioxidant and anti-inflammatory potential of BP is well documented and repeatedly reported by almost all experimental studies. A large number of studies have also confirmed, although on an experimental level, that BP has a very important potential to tackle the most important metabolic disorders that are currently known, including diabetes, obesity, and dyslipidemia. Recent research also pointed to its potential in lowering their major complications, viz. cardiovascular diseases. For the different nutritional proposes, it was emphasized that there is a major shortfall in all scientific research concerning BP. The quasi-absence of translational research on BP’s nutritional and pharmacological potential and, therefore, the quasi-absence of clinical studies, providing more robust data.

We identified numerous gaps in the available evidence due to the scoping methodology utilized. Briefly, only dozens of botanical species have been studied so far among more than one hundred thousand species that are known around the globe; providing numbers about percentages or about chemical identities is therefore very challenging and hard to admit from a scientific evidence-based point of view, especially considering the poorly representative sample that is studied from the whole earth flora. The variability will markedly hinder translational efforts and any other conclusion drawing from experimental studies. Standardization and universally consented protocols in experimental studies will be crucial as extraction methods. A great number of studies do not report the geographical and botanical origin of the used BP samples, which may deeply affect preclinical and translational significance, and therefore the clinical relevance, of reported results. There is insufficient data available about BP pharmacokinetics and pharmacodynamics. Although substantial knowledge about numerous nutrients and phytochemicals has already been acquired, understanding of how these compounds interact and act in the human body is still very poor.

Regarding the issue of safety, despite BP enjoying a long history and a wide extent of uses by humans and despite already being the main nutrient resource for honeybees, large-scale and long-period controlled clinical studies of its use, and evaluating in the “real world” context are still missing. Questions associated with the bioaccessibility and bioavailability of BP compounds in the human body still need further research.

In addition to these observations, there is a general knowledge shortfall concerning plant phyllo-spheric microbiomes which may deeply affect BP composition either in plants before pollen foraging or during the crafting of BP pellets by honeybees. These determinants of phytochemical diversity, although widely known and studied in many other natural products and phenomena, have very rarely been studied for BP.

To simplify, the essence of recommendations, from our modest point of view, is to try to resolve the gaps that we have listed above, and we recommend that future research on BP adopt the emerging high-throughput technologies such as omics sciences and computational-based simulation technics to screen the large and very heterogenous data. Stepping forward from the experimental stage to translational research will unavoidably rely on the presence of a substantial body of data, which must not only be sufficiently representative, but also sufficiently reliable, reproducible, clinically translatable, and able to greatly facilitate its clinical assessment and larger-scale adoption.

## Figures and Tables

**Table 1:** Research Protocol According to the Reporting Guidance of PARISMA-ScR Checklist for Scoping Reviews and Updated Recommendations from PRISMA 2020 Statement for Systematic Reviews.

Section	Item	Prisma-ScR Checklist Item	Reported on Page #
Title			
Title	1	Identify the report as a scoping review.	1
Abstract			
Structured summary	2	Provide a structured summary that includes (as applicable): background, objectives, eligibility criteria, sources of evidence, charting methods, results, and conclusions that relate to the review questions and objectives.	1
Introduction			
Rationale	3	Describe the rationale for the review in the context of what is already known. Explain why the review questions/objectives lend themselves to a scoping review approach.	1, 2
Objectives	4	Provide an explicit statement of the questions and objectives being addressed with reference to their key elements (e.g., population or participants, concepts, and context) or other relevant key elements used to conceptualize the review questions and/or objectives.	1, 2
Methods			
Protocol and registration	5	Indicate whether a review protocol exists; state if and where it can be accessed (e.g., a web address); and if available, provide registration information, including the registration number.	None
Eligibility criteria	6	Specify characteristics of the sources of evidence used as eligibility criteria (e.g., years considered, language, and publication status), and provide a rationale.	4, 7
Information sources	7	Describe all information sources in the search (e.g., databases with dates of coverage and contact with authors to identify additional sources), as well as the date the most recent search was executed.	7
Search	8	Present the full electronic search strategy for at least 1 database, including any limits used, such that it could be repeated.	7
Selection of sources of evidence	9	State the process for selecting sources of evidence (i.e., screening and eligibility) included in the scoping review.	4, 7
Data charting process	10	Describe the methods of charting data from the included sources of evidence (e.g., calibrated forms or forms that have been tested by the team before their use, and whether data charting was performed independently or in duplicate) and any processes for obtaining and confirming data from investigators.	3, 4, 7
Data items	11	List and define all variables for which data were sought and any assumptions and simplifications made.	2, 7–46
Critical appraisal of individual sources of evidence	12	If carried out, provide a rationale for conducting a critical appraisal of included sources of evidence; describe the methods used and how this information was used in any data synthesis (if appropriate).	None
Synthesis of results	13	Describe the methods of handling and summarizing the data that were charted.	4–7
Results			
Selection of sources of evidence	14	Give numbers of sources of evidence screened, assessed for eligibility, and included in the review, with reasons for exclusions at each stage, ideally using a flow diagram.	4,7
Characteristics of sources of evidence	15	For each source of evidence, present characteristics for which data were charted and provide the citations.	4, 7
Critical appraisal within sources of evidence	16	If carried out, present data on critical appraisal of included sources of evidence (see item 12).	35, 39, 42, 47, 48
Results of individual sources of evidence	17	For each included source of evidence, present the relevant data that were charted that relate to the review questions and objectives.	7–46
Synthesis of results	18	Summarize and/or present the charting results as they relate to the review questions and objectives.	8, 14–25, 28–33, 36, 39, 41,
Discussion			
Summary of evidence	19	Summarize the main results (including an overview of concepts, themes, and types of evidence available), link to the review questions and objectives, and consider the relevance to key groups.	28, 35, 38, 42, 45–48
Limitations	20	Discuss the limitations of the scoping review process.	48
Conclusions	21	Provide a general interpretation of the results with respect to the review questions and objectives, as well as potential implications and/or next steps.	46–48
Funding			
Funding	22	Describe sources of funding for the included sources of evidence, as well as sources of funding for the scoping review. Describe the role of the funders of the scoping review.	49

Table template from [29] Updated recommendations from PRISMA 2020 statement were retrieved from the JBI Manual for Evidence Synthesis as updated on 26 July 2022 (https://jbi-global-wiki.refined.site/space/MANUAL/4688844; accessed on 1 December 2022).

**Table 2:** BP antioxidant bioactivities: Studies published during the last four years (2019–2023).

Form of BP Use	Botanical Origin	Geographical Origin	Experiment Protocol	Reported Results	Unveiled Mechanisms	Ref.	Correlation with TPC (*)
80% ME	NA (*)	Poland	ABTS (*), DPPH (*), FRAP (*), CUPRAC (*), PCL (*)	Marked AO (*) activity (much higher than bee bread, wax and honey).	Radical scavenging, ion reduction.	[109]	Strong
Whole dried BP in water	NA	European countries	Oxidation-reduction potential (ORP)	The first study of ORP in BP. Marked improvement in AO status.	Radical scavenging. Overall decrease in oxidant abundance.	[131]	Moderate
BP fed to animal models of POS (*)	NA	NA	TAC (*) of serum measured by FRAP	TAC increased.	Radical scavenging. Apoptosis increase in ovarian cysts.	[132]	NA
BP protein hydrolysates (enzymatic)	NA	Thailand	ABTS and DPPH	AO activity, and apoptosis activation in a human lung cancer cell line.	Radical scavenging. Promoting cancer cells apoptosis.	[133]	NA
Ethanol 95% and water extracts	Multifloral: *Castanea sativa*, *Hedera helix*, *Rubus ulmifolius*.	Italy	In vitro: ABTS, DPPH, ORAC (*), FRAP, Fe^2+^. In erythrocytes: CAA (*), oxidative lysis	Good AO activities in all assays. Erythrocytes hemolysis prevention. BP microbiota yeasts also showed AO activity.	Radical scavenging, ion reduction, inhibition of free radical production, cell lysis prevention, metal-chelating ability.	[11]	NA.
Water extract	Multifloral: *Citrus aurantium*, *Apiaceae*	Morocco	DPPH, ABTS, and FRAP.	AO activity of BP was much more pronounced than honey in all assays.	Radical scavenging, ion reduction.	[134]	Strong
Absolute EE	Mainly *Brassica rapa*, *Eschscholzia californica*. Others.	Chile	FRAP, ORAC-fluorescein.	The AO activity varied significantly between samples, independently of TPC.	Radical scavenging, ion reduction.	[135]	No correlation
70% EE	Many monoflorals, Other Multiflorals	Morocco	DPPH, ABTS, Reducing Power Assay (ferric ion reduction).	Authors curiously reported that an *Ononis* Monofloral BP had the lowest activity in DPPH and ABTS test, and another botanically similar BP had the highest activity in all assays.	Radical scavenging, ion reduction.	[136]	High for DPPH and ABTS, but nil for FRAP.
BP feeding to aged horses during autumn	NA	NA	In the blood: FRAP, GSH-Px (*), [SH] (*) as indicator of OS (*).	FRAP declined significantly, and SH increase was prevented, while GSH-Px activity was not changed	Radical scavenging, protein oxidation abolition, but AO defense (GSH-Px activity) not enhanced	[137]	NA
80% ME of fermented and non-fermented BP	NA	Different European regions	In vitro: DPPH (fermentation either spontaneous or by lactic bacteria)	Increased radical scavenging activity and total phenolic and flavonoid contents in fermented BP	Radical scavenging.	[78]	High
Polysaccharide fractions of BP	*Lycium chinense*	China	In vitro: DPPH, O_2_^−^ radical scavenging, ABTS, Fe^2+^ chelating	Fraction with lower molecular weight and higher uronic acid content had the highest AO activity.	Radical scavenging, low ferrous ion chelating ability.	[138]	NA
EE	NA	Turkey	In vitro: FRAPIn vivo: Rat hippocampus	FRAP reduction in vitro and BDNF (*) increase in vivo.	Radical scavenging, AO defense enhancement.	[139]	High
80% methanol for assays, BP powder to frankfurters	Predominantly *Brassica napus* and *Fraxinus* spp.	Serbia	TAC, FRP, ABTS, DPPH, TBARS (*)	Strong AO activity in all tests, good stabilization of sausages.	Radical scavenging, ion reduction, prevention of free radial production, food stabilization	[140]	NA
Pollen feeding to zebrafish	NA	Brazil	ABTS	BP had radical scavenging activity but induced higher growth of melanoma without weight gain.	Such results have never been reported in other studies.	[141]	NA
BP feeding to broilers	NA	Saudi Arabia	DPPH, TAC, total SOD (*) (T-SOD), and CAT (*) in blood samples.	In vitro: DPPH decrease, In vivo: Increased TAC, T-SOD, and CAT activities.	Radical scavenging, AO defense enhancement.	[142]	NA
80% ethanol extract	Miscellaneous multifloral BPs	Spain	DPPH, ABTS	Botanical origin and climatic conditions determine AO activity of BP	Radical scavenging.	[9]	High
In vitro: 70% EE of wall-broken BP Animals: raw and wall-broken BP	*Rosa rugosa*	China	ORAC, DPPH, ABTS	In vitro: AO activity improved by BP wall-breaking. In vivo: Elevated SOD and CAT. MDA (*) decrease. Protection of kidney, liver, spleen and thymus.	Radical scavenging, reduction in free radical production, enhancement of AO defense, cell and tissue protection.	[143]	NA
95% EE fractionated by organic solvents	NA	Saudi Arabia	MDA levels and AO status (CAT, Vit. C, GSH), GST (*) in rat brains.	Inhibition of lipid peroxidation and increase in AO defense biomarkers depending on BP fraction. Protective effects in other organs than the brain.	Radical scavenging, AO defense enhancement, prevention of free radical generation.	[144]	Correlation present but extent not assessed
Water extract	NA	Morocco	DPPH, FRAP, Phosphomolybdate assay.	Marked DPPH decrease and ferric ion reduction.	Radical scavenging, ion reduction.	[145]	NA
70% ME	Diverse botanical species	Italy	ORAC, DPPH, ABTS	AO capacity varied with botanical species and geographical area even for the same botanical species. It was higher for multi-floral pollens.	Radical scavenging.	[107]	Significant with some exceptions
80% EE after petroleum treatment to remove lipids	Rape (*Brassica* sp.)	China	DPPH, ABTS, FRAP	AO activity decreased as follows: phenolamines fraction > crude extract > flavonoid fraction.	Radical scavenging, ion reduction.	[146]	NA
70% EE	4 Mono-floral, One bi-floral, and 13 Multifloral BPs.	South Korea	DPPH	16/18 samples had potent AO activity depended on botanical and geographical origins and climate	Radical scavenging	[105]	Absent for both TPC and TFC (*)
70% ME	NA	Poland	ABTS	AO activity varied largely between samples although always present.	Radical scavenging.	[104]	High
95% EE	Species from diverse botanical families	Turkey	TAC assessed by CERAC and CUPRAC (*).	Strong AO activity in all samples with high TPC and unsaturated fatty acid contents	Radical scavenging, ion reduction.	[147]	NA
EE	Chestnut (*Castanea* sp.)	Turkey	FRAP, CHROMAC (*), and ABTS. Fenton reaction for DNA (*) oxidative damage	Marked AO activity, protection against DNA oxidative damages.	Radical scavenging, ion reduction, Inhibition of free radical production, especially DNA oxidation byproducts	[148]	No correlation
80% EE	Multifloral (17 pollen types present at >3%)	Portugal	DPPH, Reducing Power Assay Simulated gastrointestinal digestion	Marked decrease in radical scavenging and reducing power after digestion (only one BP has the highest AO capacity at the end of the digestion).	Radical scavenging, ion reducing effect	[99]	Strong correlation
70% EE	Rape (*Brassica* sp.) BP: fermented and unfermented	China	In vitro: DPPH, ABTS, FRAP. In human hepatocytes: CAA	Both pollen types were active, but the fermented one was more potent in all assays.	Radical scavenging and reducing effect. High increase in TPC and TFC after fermentation	[70]	NA
95% EE	Mono-floral BPs.	Italy	FRAP in vitro, Cellular AO Activity in Red Blood Cells (ex vivo)	BP composition and activity varied even for similar botanical origin. Marked AO activity in both assays	Ion reduction, and cell protection against oxidative injuries.	[103]	Positive for TPC and TFC
70% EE	BP from a region with limited botanical species	Morocco	TAC evaluated by the phosphor-molybdenum method; DPPH	High AO and radical scavenging activities. TPC, TFC and TAA of BP were markedly lower than propolis	Radical scavenging and reducing effect.	[149]	NA
85% ME	Mono-floral and multifloral BPs	India	DPPH, FRAP, ABTS, Metal chelating activity (MCA) on Fe^2+^	All samples were active with variations according to the sample and used test	Radical scavenging, ion reduction and metal chelation. Similar botanical origins resulted in equivalent AO activities.	[102]	Very strong except for very few molecules
Multistep extraction by different organic solvents	*Cynara scolymus*	Serbia	DPPH, ABTS, TAC, FRAP, and FCC.	Extractable phenolic fraction largely more potent in ABTS, FRP and TAC. Lipid fraction had high TAC. Hydrolysable fraction: highest DPPH.	Radical scavenging, ion reduction, metal chelating, and inhibition of free radical production.	[150]	NA
75% EE dissolved in methanol/water, then extracted in 2 fractions: ethyl acetate and n-hexane	*Camellia japonica*	China	DPPH, FCC, FRAP, Effect on DNA Oxidation induced by Hydroxyl Radicals	In vitro: Ethyl acetate fraction had the highest TPC and AO activity, In mice liver: Increase in SOD and GSH and decrease in MDA. Microbiota modulation.	Radical scavenging, AO defense enhancement, prevention of free radical generation, Increase in the gut microbiota diversity and beneficial strain abundance.	[151]	Significant in the two studied fractions
70% EE	Multifloral samples	Romania	ABTS	Studied AO activity correlated fully with both TPC and TFC.	Radical scavenging, ion reduction.	[74]	Strong correlation
Micronized BP added to multi-flower honey	NA	Poland	TAC, DPPH, ABTS, FRAP, CUPRAC	Increase in TPC, TFC, phenolic acids, anthocyanin, and carotenoid contents of honey.	Excellent augmentation of total AO, antiradical and reducing activity of honey.	[51]	NA
Ethanol, methanol, water, and 70% ethanol extracts	*Eucalyptus marginata* and *Corymbia calophylla*	Australia	FRAP, DPPH	An extensive analysis of used extraction protocols in the literature and multiple fractions in this study.	Non-pulverized BP extracted with 70% EE coupled with agitation is the best method to maximize phenolics and AOs.	[101]	NA
Ethanol extract	NA	NA	In hypertensive rat testes: TAS (*), TOS (*), NF-κB (*), MDA, NO (*), PON1 (*) and CAT.	BP markedly restored the activity of PON1 and CAT, enhanced TAS, reduced TOS, NF-κB, and MDA levels, and relatively restored NO levels.	Reducing total oxidant status, increasing AO defense, and thus abolishing reproductive function damages.	[152]	NA
Hexane and then methanol or acidified methanol extraction	NA. Stingless bee (*Tetragonula biroi*) pot pollen.	Philippines	FRAP, DPPH, and ABTS, and lipid peroxidation inhibition (BCB (*) activity).	Significant differences between different extracts (all were active). DPPH and FRAP lower than those of *A. mellifera* BP in other studies.	Radical scavenging, ion reduction, inhibiting of free radical production (from lipid peroxidation).	[153]	High for TPC and TFC, except on lipid peroxidation
Enrichment of biscuits with dried BP	Mono-floral BP: *Brassica napus*, *Phacelia tanacetifolia* and *Helianthus annuus*	Hungary	DPPH, FRAP, TEAC (*)	Marked increases in AO activities and TPC in all samples with a ratio of 10% BP/Biscuit (*w/w*). TPC and AO activity decreased as follows: phacelia > rapeseed > sunflower	Radical scavenging, ion reduction, food stabilization.	[8]	High
70% EE	Multi-floral: *Echium plantagineum*, *Cistus ladanifer* and *Quercus* sp., etc.	Portugal	In vitro: DPPH, NO and O_2_^-^ radical assaysIn erythrocytes: HB, lipid and others; hemolysis	Radical scavenging was much lower than that of ascorbic acid.	Radical scavenging, inhibition of free radical production and intracellular reactions, cell protection against oxidation and lysis.	[154]	High, especially for selected compounds
70% EE	*Zea mays*	Malaysia	DPPH	Strong AO activities and high TPC.	Radical scavenging.	[155]	strong
BP Lipid fraction evaluated by lipidomics	*Camellia sinensis*	China	Antioxidative gene expression after DSS(*) OS, and gut barrier function	Activating the Nrf2-ARE-dependent pathway (*) and activating NF-κB, TOR (*), and Nrf2 signaling factors.	Enhancing the AO defense and the general barrier function and structure in the gut epithelium	[58]	NA
95% EE	Multi-floral: *Castanea*, *Rubus*, and *Cistus*.	Italy	Ex vivo (erythrocytes): CAA, hemolysis, cellular ROS (*) production	All samples were similarly efficient in preventing AAPH (*)-induced hemolysis and OS.	Boost of cellular AO status, prevention of membrane alteration and cell injuries and lysis.	[156]	Judged probable but not confirmed
70% EE	*Schisandra chinensis*	China	In vitro: ABTS, FRAP Infarcted rat myocardium: SOD, GSH-Px, CAT	AO activity in vitro and significant in vivo increase in AO enzyme activities at high doses of BP extract.	Radical scavenging, ion reduction, AO defense activation and myocardial tissue protection.	[157]	NA
Water extract	NA	Egypt	In Fluvastatin-induced hepatitis rat liver: MDA, GSH, GSH-Px and CAT	Lower MDA and higher GSH levels, and higher GSH-Px, and CAT activities. Synergism with thymoquinone.	Inhibition of free radical production, activation of AO defense, and liver tissue protection.	[158]	NA
Absolute EE	Diverse botanical origins	Chile	In vitro: FRAP, ORAC In a hepatic cell line: Cytotoxicity and hepatoprotection AAPH	AO activity in all samples but varied largely. Hepatic cell death prevented. Significant reduction in lipid accumulation in steatosis.	Radical scavenging, ion reduction. Cell protection against lipid loads and AAPH.	[159]	Present for TPC and TFC
BP diet feeding to *Apis mellifera* isolated in cages	Predominantly *Solidago* spp.	United States	In bee abdomens: SOD, CAT, vitellogenin (VG), HSP70 and HSP90 (*).	VG expression very high in BP-fed compared to sugar-fed bees. Other parameters did not differ.	Improvement in a vital protein status. In honeybees, VG is a vital protein with AO activity [160].	[161]	NA
Hexane, 70% ethanol and ethyl acetate successively	*Camellia sinensis*	China	NQO1 (*), Txnrd1 (*), and Nrf2 genes in colon adenocarcinoma cells	Potent increase in NQO1, Txnrd1 and Nrf2 gene expressions following the DSS-induced oxidative stress.	Enhancing cellular AO defense.	[162]	NA
70% EE	Mono-floral and multi-floral BPs	Morocco	DPPH, ABTS, FRAP	Potential AO activity with great difference in all samples, and *Thymus vulgaris* BP being the most active.	Radical scavenging, ion reduction.	[163]	No correlation
ME fractioned with hexane and dichloro-methane	*Helianthus annuus*	Thailand	DPPH	AO potential of different fractions varied from nil to low, while it was absent for crude extracts.	No AO activity. Botanical origin and extraction method may determine the AO activity.	[164]	NA
70% EE of BP from *Melipona fasciculata*	NA	Brazil	DPPH, FRAP and ABTS	AO activity present in all samples, varied largely and is higher than that of *Apis mellifera* BP in other studies.	Radical scavenging, ion reduction.	[165]	No correlation
75% EE	Rape (*Brassica* sp.)	China	In mice models of DSS-induced colitis: Oxidative and microbiota markers	Increase in SOD and GSH-Px activities decrease in NO and MPO (*) levels. Increase in microbiota diversity and beneficial strains abundance.	Radical scavenging, AO defense enhancement, prevention of free radical generation, microbiota modulation.	[166]	NA
Water or 75% EE assisted with ultrasounds or heat reflux	*Actinidia arguta*	China	In vitro: FRAP, FCC, and DPPH. In a plasmid DNA and in lymphocytes: OS-induced damages	Strong AO activities. EEs were more potent (more with ultrasound in FRAP and FCC). Potent inhibition of DNA alterations	Radical scavenging, reduction Abolishing oxidative damages to circulating DNA and cytoprotective effect on lymphocytes.	[167]	Evident (but only few samples were studied)
70% EE from pulverized BP powder	*Schisandra chinensis*	China	In H_2_O_2_-injured cardiomyocyte: Cell survival, morphology, MDA, SOD, and GSH.	Increase in cell viability, SOD and GSH, marked prevention of morphological alterations, and decrease in MDA levels.	AO defense enhancement, prevention of free radical generation, cell protection against oxidative injuries and death.	[168]	NA
Water, 80% EE and supercritical fluid extracts (SFE)	Chestnut (*Castanea* sp.)	Italy	DPPH, ferrous ion reduction.	EE by far the most active in both assays followed by SFE and water extracts. SFE was the richest in TPC.	Radical scavenging, ion reduction.	[169]	No correlation
BP powder added to skimmed goat milk	*Helianthus annuus*	Serbia	Polyphenol bio-accessibility in a digestion model. TAC, ABTS, FCC	BP reduced milk TAC and TPC, and reduced polyphenol bioaccessibility. Digestion increased RSA.	Enhancing AO potential and functionality. The TAC decrease is probably due to polyphenol capture by casein micelles.	[170]	NA
Water extracts	Different botanical origins	Egypt	DPPH	BP was the most active compared to bee bread, royal jelly, and different mixes of bee products.	Radical scavenging.	[44]	No correlation
70% EE	NA. BP of stingless bee *Scaptotrigona affinis postica*	Brazil	DPPH, ABTS, FRAP	DPPH AO activity and TPC much higher than *Apis mellifera* BP in other studies. FRAP and ABTS AO activity similar to other bee species.	Radical scavenging, ion reduction.	[171]	NA
70% ME	Multifloral BPs	Poland	ABTS	ABTS AO activity varied with TPC	Radical scavenging.	[106]	High
Water	NA	NA.	MDA, CAT and SOD in rat testes damaged by methotrexate	Decrease in MDA levels and SOD activity. No change in CAT activity. BP restored rat fertility parameters.	Reducing lipid peroxidation, enhancing AO state, and reproductive organ structure and function	[172]	NA
Water	NA. BP of stingless bee *Trigona* spp.	Indonesia	DPPH.	Mild activity: the 1/4th of vitamin C, better than propolis and honey.	Radical scavenging.	[173]	NA
70% EE	NA	Morocco	DPPH, ABTS and FRAP	AO activity in all assays. Remained however lower than vitamin C.	Radical scavenging, ion reduction, protection against induced cell and tissue injury.	[174]	NA
ME partitioned by hexane, DCM and methanol	Monofloral BPs	Thailand	DPPH, ABTS, FRAP	Only DCM fraction from *M. diplotricha* showed strong AO potential (however less than ascorbic acid).	Radical scavenging, ion reduction.	[175]	No correlation
70% EE	Monofloral *Lotus* BP	China	In vitro: GSH, SOD and MDA levels. In isoproterenol-injured cardiomyocytes: cell protection	Significant increase in GSH and SOD activity, and significant decrease in MDA levels in the studied cell line.	AO defense enhancement, prevention of free radical generation, cell protection against oxidative injuries and apoptosis.	[15]	NA

(*): [SH]: concentration of sulfhydryl groups; AAPH: 2,2′-Azobis(2-amidinopropane) dihydrochloride assay; ABTS: 2,2′-azino-bis(3-ethylbenzothiazoline-6-sulfonic acid assay; AO: antioxidant; ARE: antioxidant response element; BCB: β-carotene bleaching; BDNF: brain-derived neurotrophic factor; CAA: cellular antioxidant activity assay; CAT: catalase; CERAC: modified cerium(IV)-based antioxidant capacity assay; CHROMAC: chromium reducing antioxidant capacity assay; CUPRAC: cupric ion reducing antioxidant capacity assay; DCM: dichloromethane; DNA: deoxyribonucleic acid; DPPH: 2,2-diphenyl-1-picrylhydrazyl assay; DSS: dextran sulfate sodium; EE: ethanolic extract; FCC: ferrous ion-chelating capacity; FRAP: ferric-reducing antioxidant power; GSH-Px: glutathione peroxidase; GST: glutathione S-transferases; HB: hemoglobin; HSP: heat shock proteins; MDA: malondialdehyde; ME: methanolic extract; MPO: myeloperoxidase; NA: not available; NF-κB: nuclear factor kappa-light-chain-enhancer of activated B cells; NO: nitric oxide; NQO1: nicotinamide adenine dinucleotide phosphate (NAD(P)H) quinone dehydrogenase 1; Nrf2: Nuclear factor erythroid 2-related factor 2; ORAC: oxygen radical absorbance capacity assay; ORP: oxidation-reduction potential; OS: oxidative stress; PCL: photochemiluminescence assay; PON1: paraoxonase 1; POS: polycystic ovary syndrome; ROS: reactive oxygen species; RSA: radical scavenging activity; SOD: superoxide dismutase; TAC: total antioxidant capacity; TAS: total antioxidant status; TBARS: thiobarbituric acid reactive substance assay; TEAC: trolox equivalent antioxidant capacity; TFC: total flavonoid content; TOR: target of rapamycin; TOS: total oxidant status; TPC: total phenolic content; Txnrd1: thioredoxin reductase 1.

**Table 3:** Studies on BP anti-inflammatory activities published during the last four years.

BP Preparation	Experiment Details	Reported Results	Ref.
70% EE of unfermented and S. cerevisiae-fermented rape BP from China	In a lipopolysaccharide-treated macrophage cell line: NO, COX-2, TNF-α (*), IL-1β and IL-6 levels.	NO, COX-2, TNF-α, IL-1β, and IL-6 levels markedly decreased dose-dependently (fermented BP more potent in all tests.	[70]
95% EE of Italian BP composed mainly from *Castanea sativa* (88.8%) and *Hedera helix* sp. (4.2%).	Cell viability and gene expression of IL-8, COX-2, and ICAM-1 (*) were assessed in a TNFα-inflamed human colorectal adenocarcinoma cell line.	Pretreatment with BP extract reduced IL-8, COX-2, and ICAM-1 levels and did not alter cell viability even at very high doses.	[11]
Animals fed with BP from Turkey. Other products assessed (bee bread, honey, royal jelly and propolis).	Bee products were administered separately to rat models of chronic inflammation. Pro- and anti-inflammatory cytokines were measured in the blood.	BP increased anti-inflammatory cytokines IL-4, IL-10, and IL-1RA, and decreased pro-inflammatory cytokines TNF-α, IL-1ß, and IL-6. BP was more potent than all other bee products.	[199]
Extraction with 90% ethanol of *Fagopyrum esculentum* BP from China.	Colitis was induced by DSS in male mice. DAI and other inflammation markers (ICAM-1 expression and colon tissue imaging) were assessed.	BP extract markedly reduced DAI; corrected DSS-induced loss of weight and colon weight and length, significantly reduced spleen swelling and lymphocytes and iCAM-1 expression; corrected damages in epithelial barrier and villi, and restored AO defense and activated/regulatory T-cell balance.	[200]
Pulverized Brazilian BP was fed to zebrafish (botanical origin was not specified).	In transgenic zebrafish experimentally-induced melanoma: Metagenomic analysis of gut microbiome, and assessment of pro-inflammatory mediators in gut.	Significant increase in beneficial microbiota strains and a decrease in some known pathogenic strains. Unexpectedly, BP did not change tested pro-inflammatory mediators and induced a higher tumor growth than in control animals.	[141]
Mice models fed with 90% EE of *Fagopyrum esculentum* BP from China.	In high-fat diet- and streptozocin-induced diabetes mice: Pro- (TNF-α, IL-2 and IL-6) and anti-inflammatory (TGF-ß (*)) cytokines, and PI3K/AKT (*) in rat pancreas. Genomic study of colon microbiota.	BP reduced liver infiltration of inflammatory cells, decreased gene expression of TNF-α and TGF-ß, and increased that of PI3K and AKT, and enhanced microbiota richness, diversity, and beneficial strain abundance. Other inflammation triggers markedly reduced (e.g., OS markers, liver injuries, steatosis)	[201]
An extract of *Lotus* BP with 70% ethanol was added to a myocardial cell culture.	Cardiomyocyte hypertrophy was induced with isoproterenol.	BP inhibited the increase in pro-inflammatory cytokines (IL-6 and TNF-α) and the JAK2/STAT3 (*) signaling pathway, and increased anti-apoptosis related factors (lowered Bax and Caspases levels, and increased Bcl-2 and Bcl-2/Bax ratio (*)).	[15]
Commercial BP was fed to rats. Botanical origin not identified.	Previously PB-fed rats were treated by propionic acid to induce autistic state. Cytokines, OS markers, and gut microbiota were analyzed.	In the rat brains, BP induced marked decrease in IFN-γ (*), IL-1α, and IL-6, and had no effect on IL-12, TNF-α, and VEGF (*). BP induced a significant decrease in fecal pathogen strains and protected animals against other inflammation comorbidities such as OS and glutamate excitotoxicity.	[65]
BP, raw or fermented either spontaneously or by a selected inoculum, was used for in vitro experiments.	BP samples added either to an in vitro model of gastrointestinal batch digestion, or to a human adenocarcinoma or keratinocyte cell line cultures. Pro-inflammatory mediators, ROS, intestinal barrier function and cell viability were assessed.	Serum availability of phenolics increased by 22% with selectively fermented BP and not changed by raw and spontaneously fermented BPs. Inhibitory potential on Il-6, IL-8, MCP-1 and TNF-α was significant by all samples only in inflamed cells (selectively fermented > spontaneously fermented > raw).	[80]
Extract from Chinese *Camellia japonica* BP with 75% ethanol, dissolved in methanol and water, and then fractionated with ethyl acetate and n-hexane.	Hyperuricemic mice fed with the ethyl acetate fraction. Pro-inflammatory cytokines in serum and kidney, kidney histopathology, gut microbiota analysis and fecal SCFA (*) were assessed.	All assessed cytokines (TNF-α, IL-6, IL-1β and IL-18) in animal serum and kidneys were decreased, TLR4/MyD88/NF-κB (*) and NLRP3/ASC/Caspase-1 (*) inflammatory pathways were both inhibited, microbiota diversity was enhanced, and levels of SCFA increased. BP reduced renal histological damages and interstitial infiltration of inflammatory cells. Persistent tissular alterations in allopurinol-treated mice surprisingly disappeared in BP-treated ones.	[151]
BP water extract was administered orally to rats. Botanical and geographical origins were not indicated.	A bone marrow/spleen suppression in rats was induced by doxorubicin. BP was administered at 100 and 200 mg/kg body weight. Inflammatory cytokines, oxidative and other hematological parameters, and apoptosis-related genes were measured.	BP induced a significant decrease in PF4 (*), IL-6 and, IL-1β and a significant increase in serum GM-CSF (*), G-CSF (*), and IL-10. BP increased hematopoietic factors, and blood cells and platelet numbers, reduced OS and apoptotic mechanisms, and repaired histological injuries.	[202]
A 70% EE of BP collected by the stingless bee *Scaptotrigona affinis postica* from Brazil. Botanical Origin not indicated.	Paw edema was induced in mice either by Carrageenan (with indomethacin as standard) or Dextran (cyproheptadine as a standard). Antinociceptive effect was assessed using acetic acid or formalin.	In the acute inflammation phase, BP reduced edema volume in a more potent manner than the two drug references. It also reduced nociceptive response more markedly than indomethacin.	[171]
Methanolic extract of BP. Botanical and geographical origins not indicated.	Air pouch was induced in rats and then carrageenan was injected in it with or without BP. Morphological and molecular inflammation markers were assessed.	BP extract significantly reduced inflammatory exudate accumulation, leukocyte infiltration, granulation tissue formation, and angiogenesis. TNF-α and VEGF significantly lowered.	[203]
Purified polysaccharide fraction (BPPF) of *Fagopyrum esculentum* BP from China.	A microbiota dysbiosis was induced with ceftriaxone in mice. Effect of the BPPF on inflammatory mediators, intestinal structure and function, microbiota, and immune response were assessed.	BPPF restored blood LPS (*) and thymus and spleen atrophies to the normal values. In colonic mucosa, inflammatory cell infiltration disappeared, goblet cells and mucus area were fully restored, structural alterations were corrected, TNF-α levels were not affected, and IL-6 and IL-1β levels were fully restored. Despite some differences with control group, BPPF restored microbiota diversity, richness and corrected its balance.	[196]
Multistep and multi-solvent extracts of fresh monofloral BPs from *Camellia sinensis*, *Nelumbo nucifera*, and *Brassica campestris*.	Molecular methods, metabolomics and a macrophage cell line with LPS-induced inflammation were used to study the anti-inflammatory potential of the extracts. *C. sinensis* BP was further studied in an LPS-induced acute lung injury mouse model.	In macrophages, all extracts reduced NO release and IL-1β, IL-6, IL-10, iNOS (*), and COX-2 gene expressions, and increased HO-1 gene expressions (except *N. nucifera* BP on IL-1β, IL-6, and COX-2). MAPK (*) and NF-κB pathways were markedly suppressed by *C*. *sinensis* and *B*. *campestris* BP extracts. In all cited effects, *C*. *sinensis* BP was the most active and the richest in TPC. In the lungs, this extract reduced inflammatory cell infiltration, COX-2 and iNOS expression and NLRP3 inflammasome activation. All effects were more marked than dexamethasone used as standard.	[204]
Commercial BP bought in Saudi Arabia was fed to rats (botanical and geographical origin not indicated).	Neurotoxicity was induced in rats by prenatal exposure to methylmercury. Markers of neuroinflammation, along with other neurological parameters, were examined in rat brains after a BP diet.	Brain IFN-γ significantly reduced by BP with a greater effect obtained when mothers were fed with BP before the offspring birth. Levels of many neurotransmitters, which may interfere in an interplay with neuroinflammation, were also significantly corrected.	[34]
Chinese *Camellia sinensis* BP was treated with hexane. The residue was then treated with 70% ethanol and later with ethyl acetate.	BP extract was applied on a DSS-challenged adenocarcinoma colonic monolayer cell line. inflammatory cytokine genes expression, gut barrier function, MAPK signaling pathway were studied.	Remarkable increase in TGF-β1 and decrease in TNF-α and IL-6 gene expression. Alterations of monolayer integrity, tight junction disruption and gut permeability significantly corrected. MAPK signaling pathway was markedly suppressed.	[162]
Extract of Chinses *Schisandra chinensis* BP with 75% ethanol.	Extract was fed to obese mice models of high-fat-induced nonalcoholic fatty liver disease.	IL-1β, TNF-α, NF-κB and iNOS markedly reduced in the liver and increases in serum IL-6 and TNF-α levels were completely inhibited. Shifts in microbiota diversity and abundance were corrected.	[205]
Purified polysaccharide fractions from Chinese *Lycium chinense* BP.	DSS-challenged macrophage cell line was treated by the polysaccharide fractions. Inflammatory mediators were then assessed.	A significant increase in NO and TNF-α levels were obtained. IL-1β and IL-6 were also increased. The effects depended more tightly on the fraction type than the used dose.	[138]
Brazilian BP collected by the stingless bee *Melipona fasciculata* was extracted with 70% ethanol. Botanical origin not identified.	COX inhibition was performed in vitro. Paw edema was induced in mice either by Carrageenan (with indomethacin as standard) or Dextran (cyproheptadine as a standard). Antinociceptive effect was assessed using acetic acid or formalin.	The extract inhibited COX-1 and, in a largely more potent manner (100% inhibition with 500mg BP/kg body weight), COX-2 activity. BP extract reduced edema volume in a more potent manner than the two drug references. It also reduced nociceptive response more markedly than indomethacin.	[165]
75% EE from Chinese *Brassica* sp. BP.	Mice models of DSS-induced colitis were fed with BP extract. Histomorphometry, inflammatory mediators measurement, and microbiota analysis were performed.	Downregulation was very potent on IL-1β, and marked on IL-6, NF-κB, and IκB (*). BP extract abolished weight loss, colon shortening, spleen swelling, and villi, crypts, glandular, and epithelial alterations. The overall colitis DAI was markedly reduced. BP extract ameliorated gut microbiota diversity and the relative abundance of many beneficial genera, and completely reversed the abundance of some strains which was raised by DSS.	[166]
Hamsters were fed with BP. Botanical and geographical origin were not indicated.	BP was fed to animals after autism induction with propionic acid. Markers of neuroinflammation were evaluated in animal brain tissues. Fecal *Clostridium difficile* was searched.	BP increased IL-10, completely abolished the propionic acid-induced increase in IL-6, and significantly decreased IFN-γ, IL-1α, and VEGF. TNF-α was slightly decreased. Effect on IL-12 was insignificant. *C. difficile* toxins not detected in BP-treated animals.	[206]

(*): AKT: protein kinase B; ASC: apoptosis-associated speck-like protein; Bax: Bcl-2-associated X protein; Bcl-2: B-cell lymphoma 2; DAI: disease activity index; G-CSF: granulocyte colony-stimulating factor; GM-CSF: granulocyte-macrophage colony-stimulating factor; ICAM-1: intercellular adhesion molecule 1; IFN-γ: interferon gamma; iNOS: inducible nitric oxide synthase; IκB: i kappa B kinase; JAK2: Janus kinase 2; LPS: lipopolysaccharides; MAPK: mitogen-activated protein kinases; MyD88: myeloid differentiation primary response protein 88; NLRP3: nucleotide oligomerization domain-like receptor family, pyrin domain containing 3; PF4: platelet factor 4; PI3K: phosphatidylinositol 3-kinase; STAT3: signal transducer and activator of transcription 3; TGF-ß: transforming growth factor beta; TLR4: Toll-like receptor 4; VEGF: vascular endothelial growth factor.
